# Consequences of Exchanging Carbohydrates for Proteins in the Cholesterol Metabolism of Mice Fed a High-fat Diet

**DOI:** 10.1371/journal.pone.0049058

**Published:** 2012-11-06

**Authors:** Frédéric Raymond, Long Wang, Mireille Moser, Sylviane Metairon, Robert Mansourian, Marie-Camille Zwahlen, Martin Kussmann, Andreas Fuerholz, Katherine Macé, Chieh Jason Chou

**Affiliations:** 1 Bioanalytical Science Department, Nestlé Research Center, Lausanne, Switzerland; 2 Department of Nutrition Science and Dietetics, Syracuse University, Syracuse, New York, United States of America; 3 Proteomics and Metabonomics Core, Nestlé Institute of Health Sciences, Lausanne, Switzerland; 4 Faculty of Science, Aarhus University, Aarhus, Denmark; 5 Faculty of Life Sciences, Federal Institute of Technology, Lausanne, Switzerland; 6 Nutrition and Health Department, Nestlé Research Center, Lausanne, Switzerland; Institut Pluridisciplinaire Hubert Curien, France

## Abstract

Consumption of low-carbohydrate, high-protein, high-fat diets lead to rapid weight loss but the cardioprotective effects of these diets have been questioned. We examined the impact of high-protein and high-fat diets on cholesterol metabolism by comparing the plasma cholesterol and the expression of cholesterol biosynthesis genes in the liver of mice fed a high-fat (HF) diet that has a high (H) or a low (L) protein-to-carbohydrate (P/C) ratio. H-P/C-HF feeding, compared with L-P/C-HF feeding, decreased plasma total cholesterol and increased HDL cholesterol concentrations at 4-wk. Interestingly, the expression of genes involved in hepatic steroid biosynthesis responded to an increased dietary P/C ratio by first down-regulation (2-d) followed by later up-regulation at 4-wk, and the temporal gene expression patterns were connected to the putative activity of SREBF1 and 2. In contrast, *Cyp7a1*, the gene responsible for the conversion of cholesterol to bile acids, was consistently up-regulated in the H-P/C-HF liver regardless of feeding duration. Over expression of *Cyp7a1* after 2-d and 4-wk H-P/C-HF feeding was connected to two unique sets of transcription regulators. At both time points, up-regulation of the *Cyp7a1* gene could be explained by enhanced activations and reduced suppressions of multiple transcription regulators. In conclusion, we demonstrated that the hypocholesterolemic effect of H-P/C-HF feeding coincided with orchestrated changes of gene expressions in lipid metabolic pathways in the liver of mice. Based on these results, we hypothesize that the cholesterol lowering effect of high-protein feeding is associated with enhanced bile acid production but clinical validation is warranted. (246 words)

## Introduction

Obesity is one of the most frequently studied non-communicable diseases and excessive energy intake, reduced energy expenditure or combinations of both are related to its cause. Obesity is also associated with co-morbidities such as cardiovascular diseases, type 2 diabetes, sleep apnea, arthritis and cancers; the remission of many of which have been reported in patients after Roux-en-Y gastric bypass in morbidly obese subjects [1]. Consequently, the reduction of obesity-related morbidities is as important as the success of weight loss.

Recently, a number of clinical studies have reported rapid weight loss in obese subjects consuming low-carbohydrate diets [2], [3], [4], [5], [6], [7], [8], [9], [10]. Although the macronutrient compositions of the low-carbohydrate diets were different in each study, a proportional increase in proteins and fats was commonly found in these diets [2], [5], [7]. However, the amount of fats, especially saturated fats, in the low-carbohydrate diets promoted concerns about the risk of developing cardiovascular disease. In contrast to this belief, recent studies have demonstrated improved cholesterol profiles with HDL cholesterol concentrations being either higher or less reduced, and triglyceride concentrations being lower in obese subjects after consuming low-carbohydrate, high-fat, high-protein diets than after consuming high-carbohydrate, low-fat diets [5], [10]. However, the increase in LDL cholesterol, in spite of the concurrent elevation of HDL cholesterol, challenges the overall cardioprotective effect of low-carbohydrate high-fat diets [4].

In addition to increasing dietary fats, replacement of carbohydrates by proteins is another common feature of low-carbohydrate diets. Proteins have independent effects on plasma cholesterol concentrations. In humans, replacement of carbohydrates by proteins in fat-restricted diets resulted in rapid weight loss, reduction of plasma triglyceride and elevation of HDL cholesterol concentrations [11]. In rodents, a high-casein, high-fat diet compared with a low-casein, high-fat diet increased circulating HDL cholesterol [12]. Endo et al. reported that 11 hepatic genes involved in cholesterol metabolism were down-regulated in rats fed a protein-free diet when compared with rats fed a 12% casein diet [13]. In the same study, it was also found that a gluten diet unilaterally increased 15 cholesterol biosynthesis genes when compared with a diet containing casein [13]. In addition to gluten, soy proteins have also been demonstrated to have cholesterol-lowering benefits. Concurrent up-regulation of genes involved in steroid metabolism and reduction of serum cholesterol by soy proteins suggests a well-coordinated transcriptional regulation. Over-abundance of sterol regulatory element-binding protein 2 (SREBP2) [14] and up-regulation of 3-hydroxy-3-methylglutaryl-coenzyme reductase (HMGCR), a SREBP2 target gene and the rate-limiting enzyme for cholesterol biosynthesis [15], were reported in rodents fed a soy protein diet. Thus, available evidence indicates that both the quantity and the quality of dietary proteins can regulate the expression of genes involved in hepatic cholesterol biosynthesis. However, it has not been shown how the amount of protein in the context of high-fat and low-carbohydrate diets would affect the expression of hepatic cholesterol biosynthesis genes. Understanding the metabolic adaptations to a high-fat, high-protein diet in the liver can reveal insights for assessing the cardioprotective effects of such a diet.

In the present study, we evaluated the effect of a high-protein and high-fat diet on cholesterol metabolism by examining plasma cholesterol concentrations and the expression of cholesterol biosynthesis genes in the liver of mice fed a high-fat (HF) diet that had either a high (H) or low (L) protein-to-carbohydrate ratio (P/C). Many organs can synthesize cholesterol but the liver is the principal site for cholesterol biosynthesis, which makes it a prime target for regulating cholesterol homeostasis [16]. In addition, we measured body weight, food intake and other circulating lipids at 2 days and at 4 weeks after introduction of the dietary treatments. We expected that the changes in macronutrient composition would have a broad impact on gene expression in multiple metabolic pathways in the liver, and selected microarray analyses to monitor global gene expression to reveal metabolic adaptations of the liver to the two different high-fat diets.

## Results

### Effects of P/C ratio in a HF diet on body weight and energy intake

Forty-eight glucose intolerant diet-induced obese (DIO) mice were randomized to receive either a background L-P/C-HF diet or an experimental H-P/C-HF diet. Although it was statistically insignificant, the initial weights of mice prior to the dietary intervention were higher in the H-P/C-HF than in the L-P/C-HF group (Table 1). During the first 2 days of dietary intervention, H-P/C-HF mice consumed less calories and gained less weight than L-P/C-HF mice (Table 1). However, at sacrifice, body weight, liver, epididymal fat pad and gastrocnemius muscle weights were similar in both groups (Table 1 and S1). The suppressive effect of H-P/C-HF feeding on body weight gains was also observed after the 4-wk dietary treatment, and the weight gains were significantly less in the H-P/C-HF mice than in L-P/C-HF mice (Table 1). Energy intake was also lower in H-P/C-HF mice than L-P/C-HF mice, but the difference did not reach statistical significance (Table 1). Body weight, liver, epididymal fat pad, brown fat pad and gastrocnemius muscle weights were indistinguishable between the two groups at the end of the 4-wk treatment period (Table S1).

**Table 1:** Body weight gain and energy intake of mice fed different high-fat diets.

	*Before treatment*	*2-d after treatment*			*4-wk after treatment*		
	BW (g)	BW (g)	BW gain (g)	EI (kcal)	BW (g)	BW gain (g)	EI (kcal)
L-P/C-HF	34.2±1.2	35.0±1.1	0.75±0.10	25.5±0.8	37.8±1.2	3.63±0.43	249.3±3.8
H-P/C-HF	35.7±1.2	35.7±1.2	0.01±0.11*	21.4±0.7*	37.7±1.3	1.98±0.38*	239.1±5.1

Data are mean ± sem, n = 12/group.

* p<0.05 vs. L-P/C-HF group. BW, body weight. EI, energy intake.

### Effects of exchanging carbohydrate for protein on whole body glucose tolerance

To assess whether the ratio of dietary protein to carbohydrate affects insulin sensitivity of DIO mice, an intraperitoneal glucose tolerance test (IPGTT) was performed after the 4-wk dietary treatment. As indicated in Table 2, H-P/C-HF feeding did not affect the peak glucose value or the area under the glucose curve during the IPGTT. Baseline insulin and peak insulin levels were also comparable in the two groups (Table 2). The overnight fasting blood glucose level was the only parameter which showed a trend to be lower in H-P/C-HF than in L-P/C-HF mice (Table 2).

**Table 2:** Results of intraperitoneal glucose tolerance test (IPGTT) in mice fed the L-P/C-HF or H-P/C-HF diet for 4-wk.

	*Baseline glucose (mg/dL)*	*Peak glucose (mg/dL)*	Glucose AUC (mg/dL*min)	*Baseline insulin (ng/mL)*	*Peak insulin (ng/mL)*
L-P/C-HF	104.5±11.3	463±24.4	31807±1577	0.72±0.16	1.19±0.22
H-P/C-HF	92.5±5.6§	464±27.5	29302±2047	0.59±0.14	0.95±0.13

Data are median ± semedian, n = 12/group.

§ 0.1>p>0.05. Peak blood glucose and peak plasma insulin levels were determined at 15 minutes after the glucose challenge.

### The Diet high in P/C ratio partially improved dyslipidemia in DIO mice

**Figure 1:**
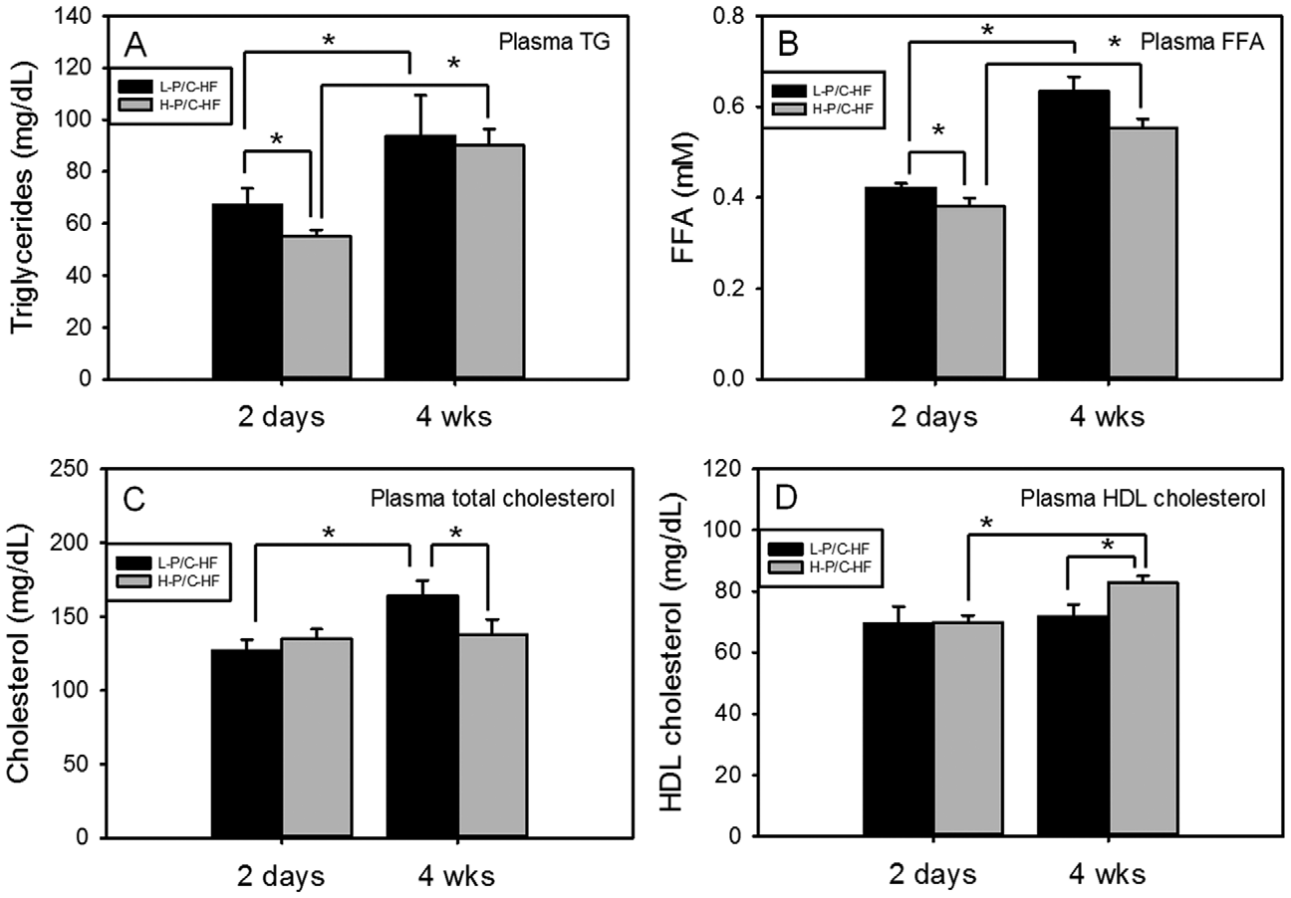
Amelioration of dyslipidemia by H-P/C-HF feeding. Plasma triglyceride (TG) (A), free fatty acids (FFA) (B), total cholesterol (C) and HDL cholesterol (D) concentrations in mice consuming the L-P/C-HF or the H-P/C-HF diet for 2-d and 4-wk. Data are median ± semedian, n = 12/group except for H-P/C-HF 2-d, n = 11. * p<0.05 by Wilcoxon test.

**Figure 2:**
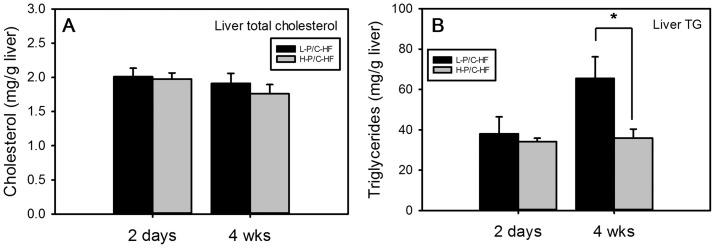
Accumulation of lipids in the liver of mice fed the L-P/C-HF or the H-P/C-HF diet. Total cholesterol (A) and triglycerides (TG) (B) in the liver of mice fed two different high-fat diets for 2-d and 4-wk. Data are median ± semedian, n = 12/group except for H-P/C-HF 2-d, n = 11. * p<0.05 by Wilcoxon test.

The plasma lipid profiles of mice fed two different HF diets were compared. After 2-d H-P/C-HF feeding, plasma triglyceride (TG) (Fig. 1A) and plasma free fatty acid (FFA) concentrations (Fig. 1B) were reduced, whereas total cholesterol (Fig. 1C) and plasma HDL cholesterol concentrations were unchanged (Fig. 1D). After 4-wk HF feeding, plasma TG and FFA concentrations increased regardless of the dietary P/C ratio (Fig. 1A and 1B). In contrast, plasma total cholesterol was lower in H-P/C-HF mice than in L-P/C-HF mice (Fig. 1C). Extending the L-P/C-HF feeding significantly increased plasma total cholesterol (from 127.01±7.17 mg/dL after 2-d to 163.96±10.60 mg/dL after 4-wk, p = 0.002), but this trend was not observed in H-P/C-HF mice (p = 0.28, Fig. 1C). Plasma HDL cholesterol displayed a different temporal pattern in response to H-P/C-HF feeding. At 2-d, the diet change had no effect on HDL cholesterol but 4-wk H-P/C-HF feeding significantly increased HDL cholesterol when compared with L-P/C-HF feeding (Fig. 1D). As a consequence, the plasma HDL cholesterol to total cholesterol ratio (HDL/total Chol) was markedly affected by both the dietary P/C ratio and feeding duration. In the L-P/C-HF group, the HDL/total Chol ratio was reduced from 53.2±4.4% after 2-d to 44.5±2.3% after 4-wk of feeding (p = 0.003), whereas the HDL/total Chol ratio in the H-P/C-HF fed mice remained similar (54.7±3.5% at 2-d vs. 63.8±5.1% at 4-wk, p = 0.178). At the end of the 4-wk study, the difference in HDL/tot Chol between the two groups was statistically significant (63.8±5.1% in H-P/C-HF group vs. 44.5±2.3% in L-P/C-HF group p<0.001). In contrast to plasma cholesterol, the liver total cholesterol concentration was not affected by the dietary P/C ratio nor by the duration of the treatment (Fig. 2A). Prolonged L-P/C-HF feeding caused accumulation of liver TG, which was prevented by H-P/C-HF feeding (Fig. 2B).

### Temporal responses of hepatic steroid metabolism genes to H-P/C-HF feeding

**Figure 3:**
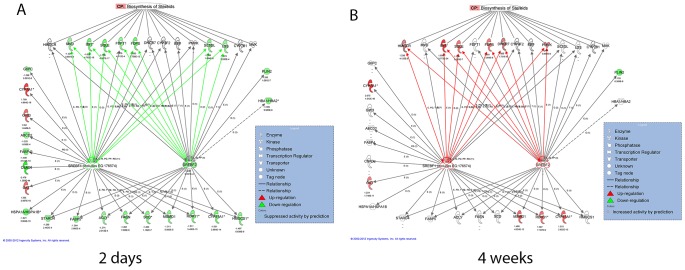
Effect of H-P/C-HF feeding on the expression of selected SREBF1 and 2 target genes. Upstream regulator analyses were performed using all of the differentially regulated genes between the two diet groups; only SREBF1 and 2 showed relevant connections to the steroid biosynthesis genes. Differentially regulated SREBF1 and 2 target genes at either time point were selected for building a relationship network, Down-regulated genes after 2-d H-P/C-HF feeding are shown in (A), and up-regulated genes after 4-wk H-P/C-HF feeding are shown in (B). Genes and the steroid biosynthesis pathway are represented as nodes. The red and green colors indicate up- and down-regulation in the H-P/C-HF compared with the L-P/C-HF group, respectively. Fold change of differentially regulated genes is indicated by the number below the gene name. Red and green lines connecting from SREBF1 and 2 to different nodes indicate positive and negative contributions, respectively. Red and green border colors of SREBF1 and 2 indicate the putative transcription activity as “active” and “inhibit”, respectively. The image was created using Ingenuity Pathways Analysis software.

To investigate if the liver was involved in the changes of plasma cholesterol levels after H-P/C-HF feeding, we performed a global gene expression analysis using Affymetrix GeneChips. Two days after the diet switch, 412 transcripts (p<0.001) were found to be differentially expressed between the H-P/C-HF and L-P/C-HF groups (Table S2). Pathway analysis of the differentially expressed genes showed that the biosynthesis of steroids pathway was the most significant differentially regulated canonical pathway in the liver of H-P/C-HF mice (Table 3) where all of the differentially regulated genes in the steroid biosynthesis pathway were down-regulated (Fig. 3A). To find out which transcription factor might be responsible for the down-regulation of steroid biosynthesis genes, we performed upstream regulator analysis with the entire dataset. Globally, AHR and NR1I3 were predicted “active” and SREBF2, SREBF1 and CEBPB were predicted “inhibit” (Table S3). Semantic relationship analysis showed that *Sqle* (−1.73), *Fdps* (−1.63), *Fdft1* (−1.28), *Mvd* (−1.22), *Lss* (−1.22), *Sc5d* (−1.34), and *Idi1* (−1.58) were direct targets of SREBF1 and 2 (Fig. 3A). In addition, the expression of *Srebf2* was significantly reduced (−1.24) in the H-P/C-HF liver, further suggesting the important role of SREBF2 in down-regulation of steroid biosynthesis genes.

**Table 3:** Top 5 differentially regulated canonical pathways between H-P/C-HF and L-P/C-HF feeding for 2-d.

Ingenuity Canonical Pathways	−log(p-value)	Molecules
Biosynthesis of Steroids	6.86E00	MVD,FDPS,SQLE,FDFT1,IDI1,LSS,SC5DL
Alanine and Aspartate Metabolism	5.05E00	ASS1, GPT, CRAT, GOT1, ASNS, AGXT, ASL
Fatty Acid Metabolism	4.72E00	ALDH1B1, Cyp2b13/Cyp2b9, ACSL3, ACADL, Cyp4a14, CYP2A6 (includes others), CYP1A2, ACOX1, CYP2B6, SDS, CYP51A1, CYP1B1
LPS/IL-1 Mediated Inhibition of RXR Function	4.52E00	ALDH1B1, ACSL3, FMO2, ACOX1, GSTA5, SLCO1A2, GSTT2/GSTT2B, Cyp4a14, CYP2A6 (includes others), FABP5, SULT1A1, CYP7A1, FABP4, CYP2B6, HMGCS1
Tryptophan Metabolism	4.4E00	AFMID, ALDH1B1, Cyp2b13/Cyp2b9, CYP2A6 (includes others), CYP1A2, CYP2B6, INMT, SDS, CYP51A1, KYNU, CYP1B1

At 4-wk, 330 transcripts were differentially regulated (p<0.001) between the two diet groups (Table S4) and the biosynthesis of steroids pathway was still one of the most differentially regulated canonical pathways in the H-P/C-HF liver (Table 4). In contrast with the 2-d results, all differentially regulated steroid biosynthesis genes (*Hmgcr*, *Idi1*, *Fdps*, *Sqle*, *Dhcr7 and Pmvk*), including *Hmgcr* - that is the gene encoding the rate limiting enzyme for cholesterol biosynthesis - were up-regulated at 4-wk..Upstream regulator analysis indicated that SREBF2, SREBF1, SIRT2, FOXO1, EGR2, PPARGC1B were predicted “active”, whereas PPARA, CEBPE, HMGA1and WT1 were predicted “inhibit” (Table S5). Putative transcriptional activities of SREBF1 and 2 coincided with the up-regulation of many steroid biosynthesis genes, which suggests the functional importance of both transcription factors in mediating the changes of steroid biosynthesis after H-P/C-HF feeding.

**Table 4:** Top 5 differentially regulated canonical pathways between H-P/C-HF and L-P/C-HF feeding for 4-wk.

Ingenuity Canonical Pathways	−log(p-value)	Molecules
Biosynthesis of Steroids	5.88E00	FDPS, SQLE, DHCR7, PMVK, IDI1, HMGCR
Complement System	5.11E00	CFD, MBL2, C1QC, C1QA, C1QB, C3AR1
Glycine, Serine and Threonine Metabolism	4.52E00	SRR, GNMT, ELOVL2, ALAS1, GOT1, GNA14, CTH, AGXT
LPS/IL-1 Mediated Inhibition of RXR Function	3.95E00	ALDH1B1, ABCG5, Cyp4a14, NR0B2, NR1I2, ACOX1, GSTA5, CYP7A1, CD14, ALAS1, FMO5, ACSL1
Fatty Acid Metabolism	3.79E00	ALDH1B1, CYP3A43, CYP1A1 (includes EG:13076), Cyp4a14, CYP4A22, ACOX1, Cyp2c70, ACSL1, CYP51A1

### Persistent up-regulation of *Cyp7a1*by H-P/C-HF feeding

**Figure 4:**
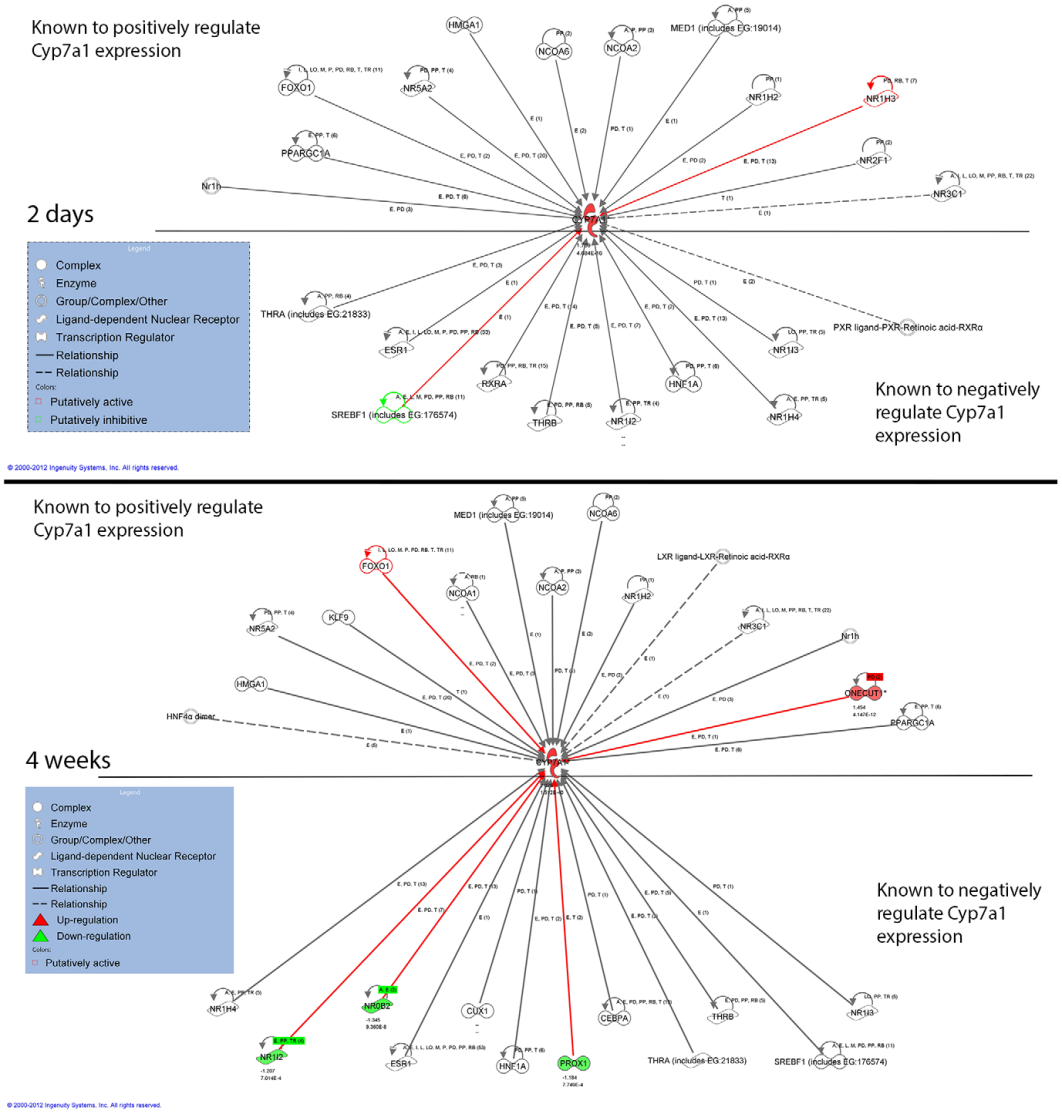
A model of Cyp7a1 regulation by H-P/C-HF feeding. Graphic illustration of a network of transcription regulators and *Cyp7a1* in the H-P/C-HF group compared with the L-P/C/HF group after 2-d (A) and 4-wk (B) of treatment. All transcription regulators that had statistically significant association with *Cyp7a1* in the present datasets were selected to build a network. Genes are represented as nodes and the expression levels of genes were overlaid on the network of nodes. Node color indicates the direction of regulation, green for down- and red for up-regulation. Fold change of differentially regulated genes is indicated by the number below gene name. Lines in between genes represent known interactions and the red color lines indicated the positive contributions. Red and green border colors of nodes indicate the putative transcription activity as “active” and “inhibit”, respectively. The image was created using IPA software.

In contrast to the temporal expression pattern of steroid biosynthesis genes, *Cyp7a1* expression was persistently up-regulated in the H-P/C-HF liver regardless of the duration of feeding (Fig. 4A and 4B). Cyp7a1 belongs to the Cyp450 family and its function is to add a hydroxyl moiety to the 7α position of cholesterol. This enzymatic activity is the rate limiting step for converting cholesterol to bile acids and is important for cholesterol homeostasis [17]. In the liver, the expression of *Cyp7a1* is transcriptionally regulated by many factors including FOXO1 [18], LRH-1 [19], LXRα [20], HNF-4α [21], Onecut1 (HNF-6) [22], NR0B2 [23], [24] and others. However, the responsible transcription regulator(s) that mediate the expression of *Cyp7a1* in the present study were not clear. To answer the question, we selected the transcription regulators that had a statistically significant relationship (p<0.05) with *Cyp7a1* gene expression from semantic relationship analyses. The transcription regulators that are known to activate or inhibit *Cyp7a1* gene expression were placed above or below *Cyp7a1*, respectively. Then, the activity of each transcription regulator was predicted according to upstream regulator analysis and the outcomes were overlaid on the existing relationships with *Cyp7a1*. Illustrated in Fig. 4A, NR1I3 (LXRα) and SREBF1 were predicted to control *Cyp7a1* gene expression after 2-d H-P/C-HF feeding, and the state of NR1I3 and SREBF1 was predicted as “active” and “inhibit” according to upstream regulator analysis, respectively. Because NR1I3 is known to positively induce *Cyp7a1* gene expression, whereas SREBF1c is known to negatively regulate it [20], [25], up-regulation of *Cyp7a1* can be explained by a direct activation by NR1I3 and reduced inhibition by SREBF1. After 4-wk of H-P/C-HF feeding, FOXO1, Onecut1, NR1I2, NR0B2 and PROX1 were predicted to have a functional relationship with *Cyp7a1* gene expression. Positive regulators including FOXO1 and Oncecut1 were predicted to be active and Onecut1 mRNA expression was up-regulated in the H-P/C-HF liver. The state of negative regulators, NR1I2 (PXR), NR0B2 (SHP) and PROX1 were predicted as “inhibit”. Altogether, bioinformatic analyses indicated that up-regulation of *Cyp7a1* gene expression after 4-wk H-P/C-HF feeding is connected to the enhanced transcriptional activation by FOXO1 and Onecut1 and the reduced transcriptional suppression by NR1I2, NR0B2 and PROX1.

## Discussion

In the present study, we investigated the effect of the protein-to-carbohydrate ratio of HF diets on cholesterol metabolism, body weight and glucose tolerance in mice. Our results agree with much of the literature demonstrating that higher amounts of dietary protein suppresses body weight gain [26], [27], [28], [29]. Also in agreement with data by Ratnayake et al. [12], our results showed that 4-wk of H-P/C-HF feeding resulted in lower plasma total cholesterol and increased HDL cholesterol. Novel findings of the present study are two folds. First, in response to the increased dietary protein-to-carbohydrate ratio, genes in the steroid biosynthesis pathway displayed complex time-dependent bi-directional expression patterns with an initial down-regulation followed by a later up-regulation. Predicted transcriptional activity of SREBF1 and 2 coincided with the expression levels of all differentially expressed steroid biosynthesis genes. Second, hepatic *Cyp7a1* gene expression was persistently up-regulated regardless of the duration of H-P/C-HF feeding, but two unique sets of transcription regulators were connected to the expression of Cyp7a1 at 2-d and 4-wk of H-P/C-HF feeding. Through the combination of physiological measurements and bioinformatic analyses of global gene expression data, our results indicate that both hepatic steroid and bile acid metabolic pathways were physiological targets of high-protein feeding. Moreover, the increased bile acid production is a novel hypothesis that should be further studied when assessing the risk of negative cardiovascular outcomes with long-term consumption of high-protein, high-fat diets.

### Effect of exchanging carbohydrate for protein on cholesterol homeostasis

One of the novel and unexpected findings of the present study is the biphasic expression pattern of steroid biosynthesis genes during the course of H-P/C-HF feeding. In response to acute or chronic H-P/C-HF feeding, SREBF1 and 2 were predicted to be the key transcription regulators. SREBFs are helix-loop-helix leucine zipper transcription factors and bind to specific *cis* elements of the target genes of which many of them are important for lipid metabolism [30]. In mammals, SREBF1 and 2 are encoded in different genes, and distinctive promoters and alternative splicing give rise to SREBF1a and SREBF1c from the SREBF1 gene [31]. SREBF1a and 1c preferentially activate the transcription of fatty acid biosynthesis genes, whereas SREBF2 mainly enhances the expression of cholesterol biosynthesis genes [32]. However, the target genes of both transcription factors are not mutually exclusive as SREBF1 and SREBF2 have been reported to regulate the expression of cholesterol and fatty acid biosynthesis genes [33], [34]. The lack of specificity of these transcription factors to steroid biosynthesis genes can explain why both SREBF1 and 2 were predicted to mediate the changes in the expression of steroid biosynthesis genes in the present study.

SREBF2-dependent cholesterol biosynthesis is controlled by complex cellular regulation, and cholesterol binding to regulatory proteins and posttranslational modifications of SREBF2 are two important steps which are required to activate SREBF2 [35]. Upon the demand of *de novo* cholesterol synthesis, inactive SREBF2 anchoring at the endoplasmic reticulum (ER) has to be processed and translocated to the nucleus to activate the transcription of target genes [36], [37]. One of the key steps governing the activation of SREBF2 is the cholesterol concentration of ER membranes. When ER cholesterol level falls below 5% of total polar lipids, the SREBF complex escorted by the SREBP cleavage-activating protein, SCAP, is transported from the ER to the Golgi apparatus [38], a key commitment step for activating SREBF2. In the present study, coordinated SREBF2 transcriptional activities and the time-dependent biphasic expression of steroid biosynthesis genes suggest that the cholesterol concentration of the ER is modified by the increased dietary protein-to-carbohydrate ratio. However, the ER cholesterol concentration was not determined and future studies are needed to validate this hypothesis.

In addition to *de novo* cholesterol biosynthesis, the balance of cellular cholesterol is regulated by uptake and export mechanisms in cells. Conversion of cholesterol to bile acids is one of the major routes for reducing cellular cholesterol concentrations and is critical for excreting cholesterol from the body [39]. Thus, the activity of Cyp7a1 that controls the rate-limiting step of bile acid biosynthesis using cholesterol as a substrate is important for maintaining whole body cholesterol homeostasis. This argument is supported by the fact that overexpression of *Cyp7a1* in mice resulted in elevated bile acid production and simultaneous reductions of plasma VLDL, IDL and LDL cholesterol and elevations in plasma HDL cholesterol [40]. In the present study, persistent up-regulation of *Cyp7a1* following H-P/C-HF feeding increased the bile acid concentration and simultaneously depleted cellular cholesterol which in the long-term could trigger the SREBF2-dependent up-regulation of cholesterol biosynthesis genes. Recently, Bortolotti et al. showed that the consumption of a high-protein, high-fat diet coincided with significant increases in circulating bile acids in humans [41], an observation that is relevant to the results of our transcriptomic analyses.

### Physiological impact of a high P/C ratio diet on body weight and glucose tolerance in mice

H-P/C-HF feeding significantly reduced body weight gains, but body weights at sacrifice were similar in both diet groups. The lack of difference in body weights is likely due to the following reasons. First, mice assigned to H-P/C-HF group were slightly heavier than control mice before feeding started. Second, the feeding duration was too short to demonstrate the effect of increasing dietary protein. In a study in which mice were fed experimental diets for 70 days, both body weight and weight gains were greater in mice fed a high-carbohydrate, high-fat diet than mice fed a low-carbohydrate, high-fat diet [42]. Third, high-protein feeding increases muscle protein synthesis [43], which may offset the weight loss effect of a high-protein diet. A future study aiming to measure the body composition of the mice after the high-protein diet feeding will be needed to confirm this hypothesis.

Recently, the effects of dietary protein on insulin sensitivity have been evaluated. In contrast to the anti-obesity effect, it remains controversial whether a high-protein diet improves insulin sensitivity. On one hand, feeding a high-protein diet for 6 months or longer induces insulin resistance in healthy non-obese individuals [44] and rats [45] leading to the argument that high-protein intake causes detrimental effects on glucose homeostasis by increasing insulin resistance [46], while on the other, eating a high-fat, high-protein diet for 5 weeks reduced plasma glucose, insulin, glucagon and triglyceride concentrations in weight stable type 2 diabetic patients [47]. The latter study is especially important because the experimental diet was high in fat and protein, and the data suggest that the levels of protein in such a diet can overcome the pathogenic nature of dietary fat and reverse insulin resistance.

In the present study, basal and peak insulin concentrations as well as plasma glucose clearance during the IPGTT were comparable in both groups, which indicate that the P/C ratio had little or no effect on glucose-stimulated insulin secretion and insulin-stimulated glucose uptake in skeletal muscle and adipose tissue under our experimental conditions. The only parameter that showed a trend to be lower after 4-wk H-P/C-HF feeding was fasting glycemia. The reduced fasting glycemia was associated with down-regulation of hepatic glucose 6 phosphatase catalytic subunit (*G6pc*) (−1.266) in the H-P/C-HF liver compared with the L-P/C-HF liver. G6PC catalyzes the dephosphorylation of glucose-6-phosphate and is the final step in glycogenolysis and *de novo* gluconeogenesis before releasing glucose into the circulation. Although increased hepatic gluconeogenesis can be independent of *G6Pc* expression [48], a high level of *G6pc* expression is found in the liver of streptozotocin-treated diabetic rats [49]. Reduced *G6pc* expression could indicate reduced hepatic glucose output. Another important step which regulates gluconeogenesis is phosphoenoylpyruvate carboxykinase (PCK1), however, *Pck1* in the liver was not differentially regulated by the diets in the present study. Hepatic steatosis is another marker that reflects liver insulin sensitivity. The amount of fat stored in the liver was significantly lower in H-P/C-HF mice than in L-P/C-HF mice. Together, our data suggest that increasing the dietary P/C ratio moderately enhances hepatic insulin sensitivity without affecting skeletal and adipose tissue insulin sensitivity, and this hypothesis should be validated using the hyperinsulinemic euglycemic clamp in a future study.

In summary, we tested whether a high-fat, high-protein diet has any effect on cholesterol metabolism. Our data demonstrate that replacement of carbohydrate by protein suppressed high-fat diet-induced hypercholesterolemia, weight gain and hepatic steatosis. By using microarray analyses, we showed that the dietary P/C ratio had time-dependent biphasic effects on the expression of steroid biosynthesis genes that are connected to the activity of SREBF1 and 2. We also identified that a high P/C ratio in a high-fat diet increased *Cyp7a1* expression and this effect was possibly orchestrated by two unique sets of transcription regulators at the acute and chronic feeding phases. More importantly, our data suggest that the cholesterol lowering effect of high-protein diets is due to enhanced bile acid production, a hypothesis that is relevant to assess the cardiovascular outcomes of high-protein high-fat feeding and should be confirmed in a long-term human study.

## Materials and Methods

### Animal experiment

The ethics of the study protocol was reviewed and approved by the Nestlé Research Center Animal Studies Committee (No. JC-05-01) and the experiments were conducted under authorization No. 1837 issued by the Service de la Consommation et des Affaires Vétérinaires (SCAV), Vaudois, Switzerland. Sixty-eight male C57BL/6J mice aged 6–7 weeks were obtained from Charles River Laboratories. They were housed individually in a room with a 12-h light-dark cycle with food (3434 Kliba AG, Kaiseraugst, Switzerland) and water *ad libitum* for 2-wk.

To induce diet-induced obesity (DIO), mice were given free access to water and a low-protein to carbohydrate ratio, high-fat diet (L-P/C-HF) with 55% energy from fat, 30% from carbohydrate, and 15% from protein (Table S6) for 10-wk. On Days 70 and 71, an intraperitoneal glucose tolerance test (IPGTT) was performed after 6-hr fasting starting at 08:00. A 30% glucose solution was injected into the intraperitoneal cavity at a dose of 2 g/kg body weight. Blood samples were collected from the tail vein at 0, 30, 60, and 90 minutes after the glucose injection. Blood glucose was determined by using an Ascensia Elite XL glucometer (Bayer AG, Zurich, Switzerland) in duplicate. Based on the results, forty-eight mice with the highest area under the glucose curve were selected for the diet intervention study on Day 72. Selected DIO mice were randomized into two groups (n = 24/group). One group of DIO mice was fed a high protein to carbohydrate ratio high-fat diet (H-P/C-HF) with 55% energy from fat, 15% from carbohydrate, and 30% from protein (Table S6). The other group of DIO mice continued eating the same L-P/C-HF diet. Twelve mice in each group were sacrificed on Day 74 (2-d feeding) and the remaining 12 mice were euthanized on Days 100 or 101 (4-wk feeding). During the diet study, body weight and food intake were monitored daily except for the weekends. On Days 93 & 94, an IPGTT was performed after a 15-hr overnight fast. The following morning, a 30% glucose solution was injected into the intraperitoneal cavity at a dose of 2 g/kg body weight. Blood samples were collected from the tail vein at 0, 15, 30, 60, and 120 minutes after glucose injection to determine glucose concentrations using the aforementioned method. Blood samples at 0, 15, and 120 minutes were also collected from the tail vein for insulin analysis. Plasma insulin concentrations were determined by Ultra Sensitive Rat Insulin ELISA Kits with mouse insulin standard (Crystal Chem Inc., Downers Grove, IL, USA) following instructions from the manufacturer. Mice were anesthetized by isoflurane (Aerrane, Baxter, Maurepas, France) before sacrifice, after which the liver, epididymal fat pads, gastrocnemius muscle, colon, and gut contents were collected and snap frozen in liquid nitrogen. Blood samples were collected in EDTA-coated tubes.

### Plasma and liver parameters

Plasma free fatty acids (FFA) were determined by an enzymatic colorimetric test using Wako NEFA C Kit (Wako Chemicals GmbH, Neuss, Germany) following the instructions provided by the manufacturer. Plasma triglycerides (TG) were determined using Triglycéride Enzymatique PAP 150 Kits (BioMérieux SA. Marcy-l'Etoile, France). Plasma total cholesterol and HDL cholesterol were measured by Wako total cholesterol and by Wako HDL cholesterol (Wako Chemicals GmbH, Neuss, Germany) kits, respectively. For determination of liver triglyceride and cholesterol concentrations, lipids in 200 mg frozen liver were extracted according to Folch et al. [50]. Liver total cholesterol (Roche diagnostics; Basel, Switzerland) and triglyceride (Roche Diagnostics; Basel; Switzerland) concentrations were quantified using commercial enzymatic colorimetric kits. For the triglyceride measurements, lipids were first hydrolyzed in a basic solution of 0.5N KOH in ethanol.

### Statistical analysis

Due to the presence of outliers, 2-ways ANOVA with interaction on the rank of the data was performed, and Wilcoxon tests were performed to assess the effect of diet and treatment duration. All data were presented as median ± semedian except for Table 1 where data were presented as mean ± sem. The semedian was computed based on the robust standard deviation: Sn of Rousseeuw. Analysis was performed with R 2.6.1: R Development Core Team (R Foundation for Statistical Computing, Vienna, Austria 2007. ISBN 3-900051-07-0, URL http://www.R-project.org).

### Microarray analysis

#### RNA extraction, sample preparation and chip processing

Liver total RNA was extracted from frozen tissue using TriPure Isolation Reagent (Roche Applied Science, Indianapolis, IN, USA), following the manufacturer's instructions. RNA samples were then purified by NucleoSpin RNA II kit (Macherey-Nagel AG, Oensingen) following the manufacturer's instructions. The quality of RNA samples was checked by using the RNA 6000 Nano Assay on the Agilent 2100 BioAnalyzer (Agilent Technologies, Inc., Palo Alto, CA, USA). Only samples with a RNA integrity number (RIN) higher than 7.5 were used for microarray analysis. For samples that did not meet the requirement, extraction and purification were repeated. Total RNA samples were stored at −80°C before microarray analysis.

All cRNA targets were synthesized, labeled, and purified according to the Affymetrix protocol. This method is based on the Eberwine T7 procedure [51]. Briefly, 5 µg of liver total RNA were used to produce double-stranded cDNA, followed by in vitro transcription, and cRNA labelling with biotin before hybridizing to the Affymetrix GeneChip mouse genome 430 2.0 chips (CA, USA), and the results were scanned by an Affymetrix GeneChip scanner 3000 7G.

#### Microarray data processing and statistical analysis

Data were normalized using the Robust Multichip Average (RMA) method. Based on the normal distribution of the datasets, the parametric Pearson's product moment correlation was applied for quality control (Figure S1A). The data matrix was further clustered in order to identify groups of potential outliers (Figure S1B). To validate the quality of datasets, a principle component analysis (PCA) followed by a leave-one-out cross-validation was applied (Figure S2, Table S7 and S8. Based on the quality test of microarray datasets, B25 was identified as an outlier, and the data was subsequently removed from the final analysis.

One-way Analysis of Variance (ANOVA) followed by Global Error Assessment (GEA) [52] was applied to discriminate the difference of hepatic gene expressions between H-P/C-HF and L-P/C-HF feeding for 2-d and 4-wk. The robust Mean Square Error (MSE) resulting from GEA was then used to calculate a new F-statistic and a robust p-value. The p-value was set at 0.001 for further analysis. The microarray data have been deposited in Gene Expression Omnibus, and the accession number is GSE20260 (http://www.ncbi.nlm.nih.gov/geo/query/acc.cgi?acc=GSE20260).

#### Ingenuity pathway analysis

Probes selected by the statistical analysis along with their differential expression value were loaded into Ingenuity Pathways Analysis software (IPA) (Ingenuity Systems, www.ingenuity.com) for annotation, redundancy checks and network and pathway analysis. The IPA Reference set was Affymetrix Mouse_430 2, and Direct and Indirect relationships were selected for analyses. Then IPA computed the data with the satisfaction of the right-tailed Fisher's Exact Test and generated significant networks based on semantic associations of these genes in metabolic or cell-signaling pathways that have been shown in published articles, books, and KEGG Ligand. For the genes with multiple probesets on the microarray, only the averaged value of fold change is reported, and individual values are shown in Table S2 and S4.

## Supporting Information

Figure S1 **Quality control of Affymetrix chips. Intensity plot of Pearson's product correlation matrix.**
(TIF)

Figure S2 **Cluster analysis of the intensity plot, whereas B25, A24, A09, and A01 were grouped in two sub-clusters, separated from the other datasets, respectively.**
(TIF)

Figure S3 **Principal component analysis (PCA) and Leave-one-out (LOO) cross validation.** The contributions to the overall variance are plotted for each putative outlier and for the first 5 components respectively.
(TIF)

Table S1 **Organ weight of mice at sacrifice.** Data are median ± SE, n = 12/group. ND, not determined.
(DOC)

Table S2 **List of regulated transcripts after H-P/C-HF feeding for 2 days.** All differentially expressed transcripts (p<0.001) resulting from a comparison of H-P/C-HF with L-P/C-HF mice after 2-d of feeding are listed. The significance of differences was estimated by a moderated ANOVA as described in the Material and Method section. The fold change with a negative and a positive value indicates down-regulation and up-regulation in the H-P/C-HF group, respectively. An Affymetrix probeset ID (Mouse 430 2.0) is provided for each gene. R in Notes represents a replicated detection with an alternative probeset for a given gene on the microarray.
(DOC)

Table S3 **Transcription factor analysis of global gene expressions at 2-d after H-P/C-HF feeding.**
(DOC)

Table S4 **List of regulated transcripts after H-P/C-HF feeding for 4 wks.** All differentially expressed transcripts (p<0.001) resulting from a comparison of H-P/C-HF with L-P/C-HF mice after 4-wk of feeding are listed. The significance of differences was estimated by a moderated ANOVA as described in the Material and Method section. The fold change with a negative and a positive value indicates down-regulation and up-regulation in the H-P/C-HF group, respectively. An Affymetrix probeset ID (Mouse 430 2.0) is provided for each gene. R in Notes represents a replicated detection with a different probeset for a given gene on the microarray.
(DOC)

Table S5 **Transcription factor analysis of global gene expressions at 4-wk after H-P/C-HF feeding.**
(DOC)

Table S6 **Nutritional composition of the diets.**
(DOC)

Table S7 **Arrays were used to perform the LOO analysis for cross validation of the PCA.**
(DOC)

Table S8 **A one-way ANOVA was applied to identify the outlier which significantly reduced the overall variability.** B25 reduced the variability down to 61.9% of the initial variance.
(DOC)

Text S1 (DOC)
