# Supplementary figures and images for: Consequences of Exchanging Carbohydrates for Proteins in the Cholesterol Metabolism of Mice Fed a High-fat Diet
Source: PLoS One. 2012 Nov 6;7(11):e49058. doi: 10.1371/journal.pone.0049058 (PMC3490911; doi:10.1371/journal.pone.0049058)

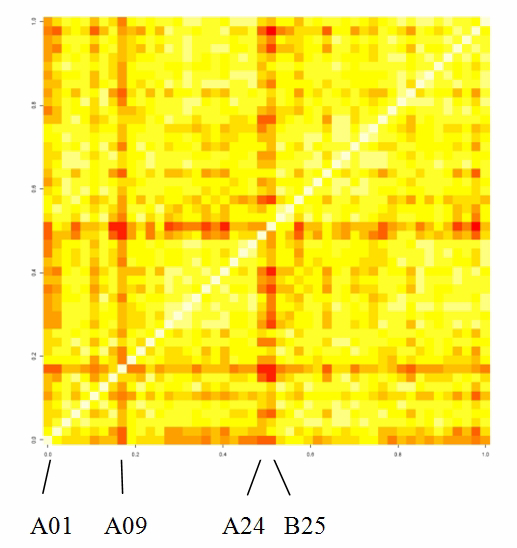

Supplement: Figure S1 — Quality control of Affymetrix chips. Intensity plot of Pearson's product correlation matrix. (TIF) [file pone.0049058.s001.tif]

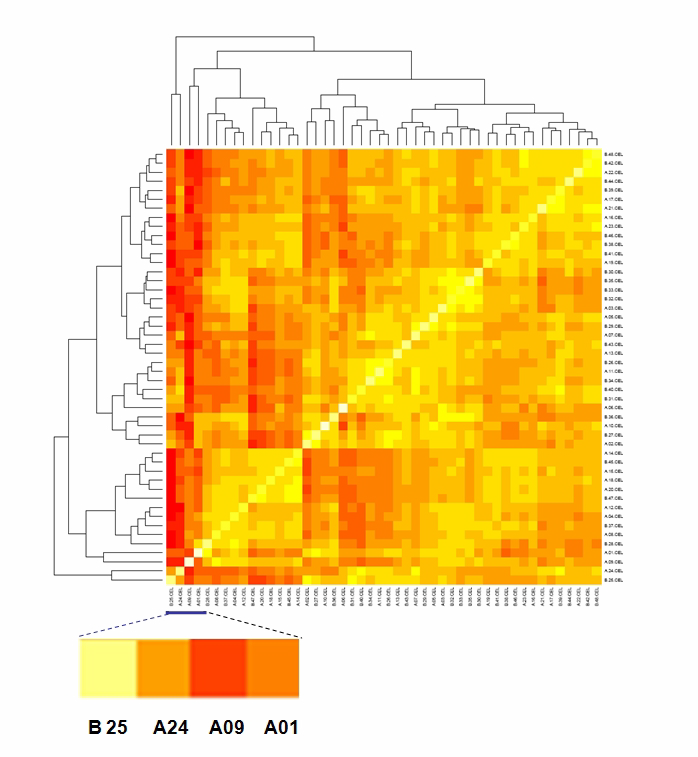

Supplement: Figure S2 — Cluster analysis of the intensity plot, whereas B25, A24, A09, and A01 were grouped in two sub-clusters, separated from the other datasets, respectively. (TIF) [file pone.0049058.s002.tif]

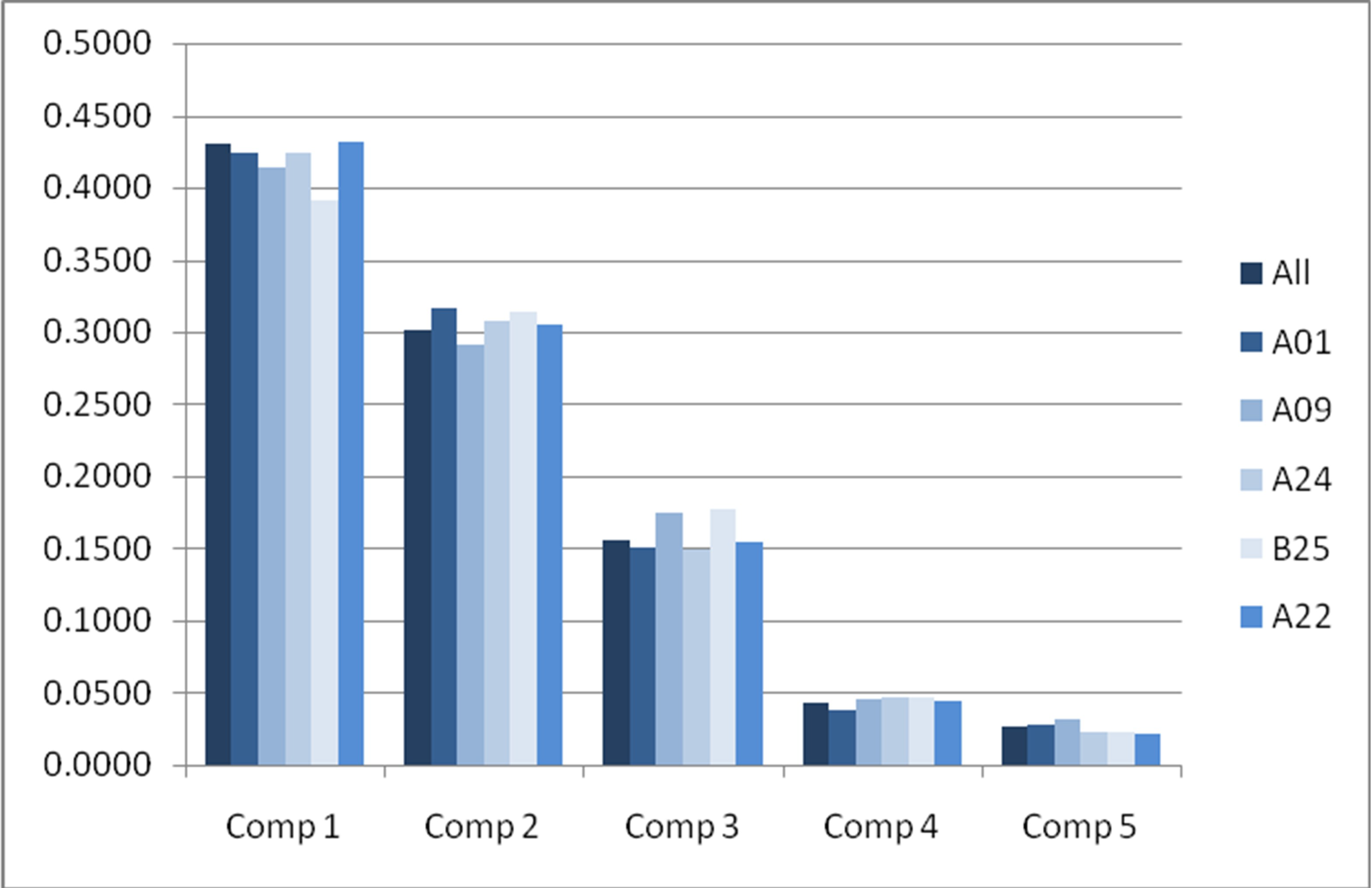

Supplement: Figure S3 — Principal component analysis (PCA) and Leave-one-out (LOO) cross validation. The contributions to the overall variance are plotted for each putative outlier and for the first 5 components respectively. (TIF) [file pone.0049058.s003.tif]
