# Supplementary material for: Consequences of Exchanging Carbohydrates for Proteins in the Cholesterol Metabolism of Mice Fed a High-fat Diet
Source: PLoS One. 2012 Nov 6;7(11):e49058. doi: 10.1371/journal.pone.0049058 (PMC3490911; doi:10.1371/journal.pone.0049058)
Supplement: Table S1 — Organ weight of mice at sacrifice. Data are median ± SE, n = 12/group. ND, not determined. (DOC) [file pone.0049058.s004.doc]

**Table S1. Organ weight of mice at sacrifice**

|  | *L-P/C-HF*  *(2 days)* | *H-P/C-HF*  *(2 days)* | *L-P/C-HF*  *(4 wks)* | *H-P/C-HF*  *(4 wks)* |
| --- | --- | --- | --- | --- |
| Liver (g) | 1.19 ± 0.05 | 1.13 ± 0.04 | 1.17 ± 0.06 | 1.23 ± 0.05 |
| Epididymal fat pad (g) | 1.72 ± 0.28 | 1.54 ± 0.2 | 2.02 ± 0.15 | 1.95 ± 0.22 |
| Brown fat pad (g) | ND | ND | 0.22 ± 0.03 | 0.17 ± 0.01 |
| Gastrocnemius muscle (g) | 0.36 ± 0.01 | 0.36 ± 0.02 | 0.40 ± 0.07 | 0.45 ± 0.06 |

Data are median ± SE, n=12/group. ND, not determined
