# Supplementary material for: Consequences of Exchanging Carbohydrates for Proteins in the Cholesterol Metabolism of Mice Fed a High-fat Diet
Source: PLoS One. 2012 Nov 6;7(11):e49058. doi: 10.1371/journal.pone.0049058 (PMC3490911; doi:10.1371/journal.pone.0049058)
Supplement: Table S2 — List of regulated transcripts after H-P/C-HF feeding for 2 days. All differentially expressed transcripts (p<0.001) resulting from a comparison of H-P/C-HF with L-P/C-HF mice after 2-d of feeding are listed. The significance of differences was estimated by a moderated ANOVA as described in the Material and Method section. The fold change with a negative and a positive value indicates down-regulation and up-regulation in the H-P/C-HF group, respectively. An Affymetrix probeset ID (Mouse 430 2.0) is provided for each gene. R in Notes represents a replicated detection with an alternative probeset for a given gene on the microarray. (DOC) [file pone.0049058.s005.doc]

Table S2. List of regulated transcripts after H-P/C-HF feeding for 2 days

| ***Gene symbol*** | ***Entrez Gene Name*** | ***Fold Change compared to L-P/C-HF*** | ***p-value*** | ***ProbeSet ID*** | ***Notes*** |
| --- | --- | --- | --- | --- | --- |
| CSAD | cysteine sulfinic acid decarboxylase | -2.478 | 1.39E-44 | 1427981_a_at |  |
| DNASE2 | deoxyribonuclease II, lysosomal | -2.445 | 4.09E-47 | 1448986_x_at | R |
| SAA1 | serum amyloid A1 | -1.792 | 5.05E-18 | 1450826_a_at |  |
| SQLE | squalene epoxidase | -1.726 | 4.31E-17 | 1415993_at |  |
| Unknown | Unknown | -1.672 | 4.70E-18 | 1443147_at |  |
| DYNLL1 | dynein, light chain, LC8-type 1 | -1.633 | 3.43E-13 | 1417339_a_at | R |
| FDPS | farnesyl diphosphate synthase (farnesyl pyrophosphate synthetase, dimethylallyltranstransferase, geranyltranstransferase) | -1.630 | 7.76E-13 | 1423418_at |  |
| IDI1 | isopentenyl-diphosphate delta isomerase 1 | -1.626 | 4.76E-16 | 1423804_a_at | R |
| HSPA1B | heat shock 70kDa protein 1B | -1.621 | 3.90E-18 | 1427126_at | R |
| HSPA1B | heat shock 70kDa protein 1B | -1.612 | 2.00E-15 | 1427127_x_at | R |
| A2M | alpha-2-macroglobulin | -1.589 | 2.88E-17 | 1434719_at |  |
| C730029A08RIK | RIKEN cDNA C730029A08 gene | -1.570 | 2.18E-10 | 1436169_at | R |
| 9130221J18RIK | RIKEN cDNA 9130221J18 gene | -1.568 | 7.46E-15 | 1446368_at |  |
| HSPA1B | heat shock 70kDa protein 1B | -1.562 | 7.23E-12 | 1452318_a_at | R |
| SLC41A2 | solute carrier family 41, member 2 | -1.556 | 1.79E-10 | 1452445_at |  |
| DYNLL1 | dynein, light chain, LC8-type 1 | -1.553 | 2.09E-10 | 1456125_a_at | R |
| Unknown | Unknown | -1.551 | 1.13E-10 | 1437073_x_at |  |
| CYP51A1 | cytochrome P450, family 51, subfamily A, polypeptide 1 | -1.551 | 2.66E-14 | 1422533_at | R |
| C730029A08RIK | RIKEN cDNA C730029A08 gene | -1.548 | 1.74E-10 | 1436168_at | R |
| FOS | FBJ murine osteosarcoma viral oncogene homolog | -1.547 | 7.43E-13 | 1423100_at |  |
| DYNLL1 | dynein, light chain, LC8-type 1 | -1.542 | 5.25E-12 | 1448682_at | R |
| ID3 | inhibitor of DNA binding 3, dominant negative helix-loop-helix protein | -1.539 | 1.47E-12 | 1416630_at |  |
| IDI1 | isopentenyl-diphosphate delta isomerase 1 | -1.536 | 1.35E-10 | 1451122_at | R |
| PAQR9 | progestin and adipoQ receptor family member IX | -1.520 | 7.80E-10 | 1455025_at |  |
| NUCB2 | nucleobindin 2 | -1.519 | 7.43E-14 | 1418355_at |  |
| Unknown | Unknown | -1.512 | 8.56E-10 | 1433898_at |  |
| RDH11 | retinol dehydrogenase 11 (all-trans/9-cis/11-cis) | -1.511 | 5.48E-13 | 1418760_at | R |
| HMGCS1 | 3-hydroxy-3-methylglutaryl-Coenzyme A synthase 1 (soluble) | -1.497 | 6.64E-09 | 1433444_at | R |
| CP | ceruloplasmin (ferroxidase) | -1.496 | 1.03E-10 | 1441326_at | R |
| SOCS3 | suppressor of cytokine signaling 3 | -1.478 | 7.52E-09 | 1455899_x_at | R |
| SOCS3 | suppressor of cytokine signaling 3 | -1.472 | 1.61E-10 | 1456212_x_at | R |
| SERPINA4-PS1 | serine (or cysteine) peptidase inhibitor, clade A, member 4, pseudogene 1 | -1.471 | 2.60E-08 | 1444297_at | R |
| FASN | fatty acid synthase | -1.462 | 1.53E-08 | 1423828_at |  |
| SCD | stearoyl-CoA desaturase (delta-9-desaturase) | -1.460 | 1.19E-07 | 1415965_at | R |
| SERPINA4-PS1 | serine (or cysteine) peptidase inhibitor, clade A, member 4, pseudogene 1 | -1.458 | 7.48E-08 | 1444296_a_at | R |
| HMGCS1 | 3-hydroxy-3-methylglutaryl-Coenzyme A synthase 1 (soluble) | -1.454 | 5.50E-08 | 1433443_a_at | R |
| ME1 | malic enzyme 1, NADP(+)-dependent, cytosolic | -1.454 | 3.60E-08 | 1430307_a_at | R |
| DERL3 | Der1-like domain family, member 3 | -1.450 | 3.46E-08 | 1453677_a_at |  |
| Unknown | Unknown | -1.448 | 1.27E-09 | 1426936_at |  |
| HMGCS1 | 3-hydroxy-3-methylglutaryl-Coenzyme A synthase 1 (soluble) | -1.445 | 2.42E-07 | 1433445_x_at | R |
| FABP4 | fatty acid binding protein 4, adipocyte | -1.430 | 3.51E-10 | 1417023_a_at | R |
| SLC25A30 | solute carrier family 25, member 30 | -1.425 | 6.37E-07 | 1450018_s_at | R |
| ISYNA1 | inositol-3-phosphate synthase 1 | -1.423 | 1.05E-09 | 1415977_at |  |
| LCN2 | lipocalin 2 | -1.412 | 9.77E-07 | 1427747_a_at |  |
| GPD1 | glycerol-3-phosphate dehydrogenase 1 (soluble) | -1.410 | 6.36E-07 | 1448249_at | R |
| SERPINA4-PS1 | serine (or cysteine) peptidase inhibitor, clade A, member 4, pseudogene 1 | -1.408 | 3.83E-07 | 1448092_x_at | R |
| CXCL2 | chemokine (C-X-C motif) ligand 2 | -1.404 | 4.98E-07 | 1419209_at | R |
| SLC30A10 | solute carrier family 30, member 10 | -1.404 | 2.47E-08 | 1438751_at | R |
| CRELD2 | cysteine-rich with EGF-like domains 2 | -1.403 | 1.00E-06 | 1452754_at |  |
| HMGCS1 | 3-hydroxy-3-methylglutaryl-Coenzyme A synthase 1 (soluble) | -1.400 | 5.38E-07 | 1433446_at | R |
| CYP51A1 | cytochrome P450, family 51, subfamily A, polypeptide 1 | -1.399 | 5.84E-07 | 1450646_at | R |
| FKBP11 | FK506 binding protein 11, 19 kDa | -1.393 | 1.84E-06 | 1417267_s_at |  |
| SLC25A30 | solute carrier family 25, member 30 | -1.392 | 5.44E-06 | 1420836_at | R |
| SLC25A30 | solute carrier family 25, member 30 | -1.392 | 4.31E-08 | 1425948_a_at | R |
| ASNS | asparagine synthetase | -1.391 | 4.62E-11 | 1433966_x_at |  |
| SLC25A30 | solute carrier family 25, member 30 | -1.390 | 1.29E-06 | 1420835_at | R |
| TUBB2A | tubulin, beta 2A | -1.389 | 1.18E-06 | 1427347_s_at |  |
| OSGIN1 | oxidative stress induced growth inhibitor 1 | -1.388 | 3.24E-06 | 1424022_at |  |
| TSC22D1 | TSC22 domain family, member 1 | -1.387 | 5.15E-06 | 1425742_a_at | R |
| BPGM | 2,3-bisphosphoglycerate mutase | -1.387 | 1.27E-09 | 1415864_at |  |
| SAA2 | serum amyloid A2 | -1.386 | 2.11E-06 | 1449326_x_at | R |
| FABP4 | fatty acid binding protein 4, adipocyte | -1.385 | 8.82E-09 | 1451263_a_at | R |
| CPNE8 | copine VIII | -1.381 | 1.63E-09 | 1430520_at | R |
| PPAPDC1B | phosphatidic acid phosphatase type 2 domain containing 1B | -1.381 | 5.26E-07 | 1428154_s_at |  |
| SLC13A3 | solute carrier family 13 (sodium-dependent dicarboxylate transporter), member 3 | -1.379 | 1.31E-07 | 1438377_x_at | R |
| CXCL2 | chemokine (C-X-C motif) ligand 2 | -1.376 | 3.42E-06 | 1457644_s_at | R |
| NRP1 | neuropilin 1 | -1.376 | 3.10E-06 | 1448943_at | R |
| CRAT | carnitine acetyltransferase | -1.373 | 1.26E-07 | 1417008_at |  |
| NRP1 | neuropilin 1 | -1.371 | 5.66E-06 | 1418084_at | R |
| C5ORF28 | chromosome 5 open reading frame 28 | -1.368 | 3.07E-08 | 1426607_at |  |
| COL27A1 | collagen, type XXVII, alpha 1 | -1.367 | 1.58E-06 | 1429549_at | R |
| VNN1 | vanin 1 | -1.367 | 4.12E-06 | 1447845_s_at | R |
| RAD51L1 | RAD51-like 1 (S. cerevisiae) | -1.366 | 2.84E-08 | 1421430_at |  |
| HBA2 | hemoglobin, alpha 2 | -1.365 | 9.98E-08 | 1417714_x_at | R |
| RETSAT | retinol saturase (all-trans-retinol 13,14-reductase) | -1.364 | 8.29E-06 | 1424716_at | R |
| CD9 | CD9 molecule | -1.364 | 8.07E-07 | 1416066_at |  |
| SERPINA7 | serpin peptidase inhibitor, clade A (alpha-1 antiproteinase, antitrypsin), member 7 | -1.364 | 5.15E-06 | 1438617_at |  |
| SC5DL | sterol-C5-desaturase (ERG3 delta-5-desaturase homolog, S. cerevisiae)-like | -1.359 | 1.69E-06 | 1434520_at |  |
| ARPC1B | actin related protein 2/3 complex, subunit 1B, 41kDa | -1.358 | 4.23E-07 | 1416226_at |  |
| PCSK9 | proprotein convertase subtilisin/kexin type 9 | -1.356 | 4.62E-08 | 1437453_s_at |  |
| RAB30 | RAB30, member RAS oncogene family | -1.355 | 1.68E-07 | 1426452_a_at |  |
| ORM1 | orosomucoid 1 | -1.354 | 4.63E-07 | 1420438_at |  |
| GPD1 | glycerol-3-phosphate dehydrogenase 1 (soluble) | -1.353 | 1.53E-05 | 1416204_at | R |
| B3GALT1 | UDP-Gal:betaGlcNAc beta 1,3-galactosyltransferase, polypeptide 1 | -1.352 | 3.34E-06 | 1460509_at |  |
| APCS | amyloid P component, serum | -1.347 | 6.45E-06 | 1419059_at |  |
| ERO1LB | ERO1-like beta (S. cerevisiae) | -1.346 | 1.93E-05 | 1434714_at | R |
| NFIL3 | nuclear factor, interleukin 3 regulated | -1.345 | 3.62E-07 | 1418932_at |  |
| GALE | UDP-galactose-4-epimerase | -1.344 | 7.14E-07 | 1424140_at |  |
| GAS6 | growth arrest-specific 6 | -1.342 | 4.02E-06 | 1417399_at |  |
| LHX6 | LIM homeobox 6 | -1.340 | 7.51E-07 | 1425094_a_at | R |
| NRP1 | neuropilin 1 | -1.338 | 2.45E-06 | 1448944_at | R |
| C12ORF72 | chromosome 12 open reading frame 72 | -1.338 | 1.73E-05 | 1427202_at |  |
| MOXD1 | monooxygenase, DBH-like 1 | -1.338 | 1.54E-07 | 1422643_at |  |
| ID2 | inhibitor of DNA binding 2, dominant negative helix-loop-helix protein | -1.337 | 3.79E-05 | 1435176_a_at | R |
| TMEM141 | transmembrane protein 141 | -1.336 | 5.45E-08 | 1435258_at | R |
| H3F3C | H3 histone, family 3C | -1.336 | 1.03E-06 | 1430357_at |  |
| NRP1 | neuropilin 1 | -1.334 | 1.29E-05 | 1457198_at | R |
| ACOX1 | acyl-Coenzyme A oxidase 1, palmitoyl | -1.334 | 3.08E-05 | 1444518_at |  |
| ERO1LB | ERO1-like beta (S. cerevisiae) | -1.333 | 1.90E-05 | 1425705_a_at | R |
| VNN1 | vanin 1 | -1.333 | 1.34E-05 | 1418486_at | R |
| Unknown | Unknown | -1.330 | 4.33E-07 | 1435628_x_at |  |
| HBD | hemoglobin, delta | -1.329 | 1.04E-06 | 1417184_s_at |  |
| Unknown | Unknown | -1.327 | 2.52E-07 | 1447108_at |  |
| GPD2 | glycerol-3-phosphate dehydrogenase 2 (mitochondrial) | -1.327 | 3.33E-05 | 1417434_at | R |
| SLC30A10 | solute carrier family 30, member 10 | -1.327 | 7.33E-06 | 1439934_at | R |
| ID2 | inhibitor of DNA binding 2, dominant negative helix-loop-helix protein | -1.326 | 2.42E-06 | 1453596_at | R |
| ACOT1 | acyl-CoA thioesterase 1 | -1.326 | 5.10E-05 | 1422925_s_at |  |
| GBP6 | guanylate binding protein family, member 6 | -1.322 | 8.62E-06 | 1447927_at | R |
| ME1 | malic enzyme 1, NADP(+)-dependent, cytosolic | -1.319 | 3.63E-05 | 1416632_at | R |
| MAGED1 | melanoma antigen family D, 1 | -1.319 | 3.89E-05 | 1450062_a_at |  |
| PHXR5 | per-hexamer repeat gene 5 | -1.318 | 3.95E-06 | 1422320_x_at |  |
| HSPA13 | heat shock protein 70kDa family, member 13 | -1.316 | 2.52E-05 | 1433772_at | R |
| CP | ceruloplasmin (ferroxidase) | -1.314 | 3.80E-05 | 1417496_at | R |
| SOCS3 | suppressor of cytokine signaling 3 | -1.314 | 1.68E-05 | 1416576_at | R |
| BMP1 | bone morphogenetic protein 1 | -1.312 | 4.10E-07 | 1427457_a_at |  |
| TSC22D1 | TSC22 domain family, member 1 | -1.311 | 5.91E-05 | 1454971_x_at | R |
| REG3G | regenerating islet-derived 3 gamma | -1.311 | 7.28E-07 | 1416297_s_at |  |
| SC4MOL | sterol-C4-methyl oxidase-like | -1.311 | 5.00E-05 | 1423078_a_at |  |
| HSPA13 | heat shock protein 70kDa family, member 13 | -1.308 | 9.87E-06 | 1429502_at | R |
| AA408650 | expressed sequence AA408650 | -1.308 | 2.65E-06 | 1440221_at |  |
| SERPINA12 | serpin peptidase inhibitor, clade A (alpha-1 antiproteinase, antitrypsin), member 12 | -1.307 | 1.72E-05 | 1421092_at |  |
| DNASE2 | deoxyribonuclease II, lysosomal | -1.306 | 3.59E-06 | 1430135_at | R |
| Unknown | Unknown | -1.304 | 2.80E-05 | 1447340_at |  |
| IFI27L2B | interferon, alpha-inducible protein 27 like 2B | -1.304 | 1.00E-05 | 1425120_x_at |  |
| GUK1 | guanylate kinase 1 | -1.302 | 9.14E-05 | 1416395_at |  |
| PAPSS1 | 3'-phosphoadenosine 5'-phosphosulfate synthase 1 | -1.301 | 5.67E-05 | 1415890_at |  |
| GMDS | GDP-mannose 4,6-dehydratase | -1.300 | 4.63E-06 | 1434158_at | R |
| HIST1H4E | histone cluster 1, H4e | -1.299 | 1.81E-06 | 1424854_at |  |
| HSD17B7 | hydroxysteroid (17-beta) dehydrogenase 7 | -1.298 | 1.68E-04 | 1457248_x_at | R |
| 2610305J24RIK | hypothetical gene LOC72520 | -1.298 | 4.28E-06 | 1428670_at |  |
| ST6GAL1 | ST6 beta-galactosamide alpha-2,6-sialyltranferase 1 | -1.298 | 2.91E-04 | 1420928_at |  |
| AKR1B1 | aldo-keto reductase family 1, member B1 (aldose reductase) | -1.297 | 7.66E-07 | 1456590_x_at |  |
| HSD17B7 | hydroxysteroid (17-beta) dehydrogenase 7 | -1.296 | 2.53E-05 | 1417871_at | R |
| CDA | cytidine deaminase | -1.296 | 5.29E-06 | 1427357_at |  |
| PGD | phosphogluconate dehydrogenase | -1.295 | 9.90E-05 | 1437380_x_at | R |
| LONP2 | lon peptidase 2, peroxisomal | -1.294 | 5.43E-06 | 1436655_at |  |
| PGD | phosphogluconate dehydrogenase | -1.293 | 1.08E-04 | 1436771_x_at | R |
| GIMAP9 | GTPase, IMAP family member 9 | -1.291 | 1.45E-06 | 1437756_at |  |
| EGR1 | early growth response 1 | -1.287 | 1.27E-04 | 1417065_at |  |
| GT(ROSA)26SOR | gene trap ROSA 26, Philippe Soriano | -1.287 | 7.81E-06 | 1452239_at |  |
| Unknown | Unknown | -1.285 | 1.79E-04 | 1445583_x_at |  |
| REEP5 | receptor accessory protein 5 | -1.285 | 1.53E-04 | 1419398_a_at | R |
| PLIN2 | perilipin 2 | -1.285 | 1.09E-07 | 1448318_at |  |
| SLC10A2 | solute carrier family 10 (sodium/bile acid cotransporter family), member 2 | -1.285 | 4.97E-07 | 1450245_at |  |
| SPP1 | secreted phosphoprotein 1 | -1.285 | 2.46E-04 | 1449254_at |  |
| NELF | nasal embryonic LHRH factor | -1.283 | 3.15E-04 | 1436959_x_at |  |
| ARSG | arylsulfatase G | -1.282 | 4.41E-05 | 1452277_at |  |
| HLA-DQA1 | major histocompatibility complex, class II, DQ alpha 1 | -1.282 | 2.99E-04 | 1438858_x_at |  |
| TMED9 | transmembrane emp24 protein transport domain containing 9 | -1.282 | 1.34E-04 | 1439448_x_at |  |
| ACSL3 | acyl-CoA synthetase long-chain family member 3 | -1.281 | 2.09E-05 | 1452771_s_at |  |
| HBA2 | hemoglobin, alpha 2 | -1.280 | 2.63E-07 | 1428361_x_at | R |
| SEC11C | SEC11 homolog C (S. cerevisiae) | -1.280 | 4.51E-04 | 1460698_a_at |  |
| GALNT2 | UDP-N-acetyl-alpha-D-galactosamine:polypeptide N-acetylgalactosaminyltransferase 2 (GalNAc-T2) | -1.279 | 2.93E-04 | 1426756_at |  |
| TSC22D1 | TSC22 domain family, member 1 | -1.278 | 2.62E-04 | 1454758_a_at | R |
| FDFT1 | farnesyl-diphosphate farnesyltransferase 1 | -1.278 | 4.11E-04 | 1438322_x_at |  |
| MVP | major vault protein | -1.278 | 3.39E-05 | 1448618_at |  |
| TMEM167A | transmembrane protein 167A | -1.278 | 3.12E-04 | 1450904_at |  |
| SLC38A10 | solute carrier family 38, member 10 | -1.277 | 3.69E-05 | 1429979_a_at | R |
| FAM25A | family with sequence similarity 25, member A | -1.277 | 4.02E-04 | 1437019_at |  |
| ACLY | ATP citrate lyase | -1.274 | 2.91E-04 | 1451666_at | R |
| DAK | dihydroxyacetone kinase 2 homolog (S. cerevisiae) | -1.274 | 8.35E-04 | 1425300_at |  |
| SLC38A10 | solute carrier family 38, member 10 | -1.273 | 3.89E-04 | 1427294_a_at | R |
| G0S2 | G0/G1switch 2 | -1.272 | 5.08E-04 | 1448700_at |  |
| CYP2D40 | cytochrome P450, family 2, subfamily d, polypeptide 40 | -1.271 | 1.65E-04 | 1430814_at |  |
| TUBA1B | tubulin, alpha 1b | -1.270 | 6.01E-04 | 1423846_x_at |  |
| ACLY | ATP citrate lyase | -1.268 | 3.13E-04 | 1439459_x_at | R |
| CPNE8 | copine VIII | -1.268 | 1.66E-05 | 1430521_s_at | R |
| PGD | phosphogluconate dehydrogenase | -1.267 | 1.20E-04 | 1423706_a_at | R |
| GPR172B | G protein-coupled receptor 172B | -1.267 | 3.02E-05 | 1439451_x_at |  |
| INHBA | inhibin, beta A | -1.267 | 1.14E-04 | 1422053_at |  |
| TMEM56 | transmembrane protein 56 | -1.266 | 6.66E-05 | 1456718_at | R |
| G6PC | glucose-6-phosphatase, catalytic subunit | -1.266 | 9.58E-05 | 1417880_at |  |
| TMEM64 | transmembrane protein 64 | -1.266 | 7.92E-04 | 1433735_a_at |  |
| RRBP1 | ribosome binding protein 1 homolog 180kDa (dog) | -1.265 | 8.53E-04 | 1426123_a_at | R |
| MANF | mesencephalic astrocyte-derived neurotrophic factor | -1.265 | 8.16E-04 | 1428112_at |  |
| STARD4 | StAR-related lipid transfer (START) domain containing 4 | -1.265 | 2.46E-04 | 1429239_a_at |  |
| FABP5 | fatty acid binding protein 5 (psoriasis-associated) | -1.264 | 7.44E-04 | 1416021_a_at | R |
| FABP5 | fatty acid binding protein 5 (psoriasis-associated) | -1.264 | 2.66E-04 | 1416022_at | R |
| CTSC | cathepsin C | -1.264 | 2.10E-06 | 1437939_s_at |  |
| EVL | Enah/Vasp-like | -1.264 | 5.06E-06 | 1434920_a_at |  |
| HAPLN4 | hyaluronan and proteoglycan link protein 4 | -1.264 | 6.22E-05 | 1435650_at |  |
| RRBP1 | ribosome binding protein 1 homolog 180kDa (dog) | -1.263 | 9.13E-04 | 1449221_a_at | R |
| EPHX1 | epoxide hydrolase 1, microsomal (xenobiotic) | -1.263 | 2.93E-04 | 1422438_at |  |
| TMEM141 | transmembrane protein 141 | -1.261 | 1.31E-04 | 1435259_s_at | R |
| AI987986 | expressed sequence AI987986 | -1.261 | 1.30E-04 | 1457717_at |  |
| LOC729756 | similar to hCG2041108 | -1.260 | 5.18E-05 | 1447729_s_at |  |
| TARS | threonyl-tRNA synthetase | -1.260 | 5.72E-04 | 1460323_at |  |
| Unknown | Unknown | -1.259 | 2.29E-05 | 1440824_at |  |
| C4ORF34 | chromosome 4 open reading frame 34 | -1.259 | 6.45E-04 | 1435464_at |  |
| INHBC | inhibin, beta C | -1.259 | 8.67E-04 | 1422001_at |  |
| TOR2A | torsin family 2, member A | -1.259 | 7.72E-05 | 1438547_x_at |  |
| TMSB10 | thymosin beta 10 | -1.258 | 1.07E-04 | 1436902_x_at |  |
| Unknown | Unknown | -1.256 | 3.68E-05 | 1447285_at |  |
| DNAJC3 | DnaJ (Hsp40) homolog, subfamily C, member 3 | -1.256 | 7.70E-04 | 1419163_s_at |  |
| TMEM56 | transmembrane protein 56 | -1.255 | 1.15E-04 | 1439353_x_at | R |
| UBA5 | ubiquitin-like modifier activating enzyme 5 | -1.255 | 2.15E-04 | 1435247_at |  |
| DNAJC12 | DnaJ (Hsp40) homolog, subfamily C, member 12 | -1.253 | 5.61E-04 | 1417441_at |  |
| UBE2L6 | ubiquitin-conjugating enzyme E2L 6 | -1.253 | 7.69E-05 | 1417172_at |  |
| Unknown | Unknown | -1.252 | 1.95E-06 | 1438313_at |  |
| SSR1 | signal sequence receptor, alpha | -1.252 | 3.62E-05 | 1441327_a_at | R |
| GAS2L1 | growth arrest-specific 2 like 1 | -1.252 | 1.36E-04 | 1434928_at |  |
| RAI14 | retinoic acid induced 14 | -1.252 | 7.52E-04 | 1417400_at |  |
| ATP11C | ATPase, class VI, type 11C | -1.251 | 1.40E-04 | 1442367_at |  |
| MAL2 | mal, T-cell differentiation protein 2 | -1.251 | 8.38E-04 | 1427042_at |  |
| UGGT1 | UDP-glucose glycoprotein glucosyltransferase 1 | -1.251 | 1.21E-04 | 1448009_at |  |
| HACL1 | 2-hydroxyacyl-CoA lyase 1 | -1.250 | 8.64E-04 | 1449047_at |  |
| LASP1 | LIM and SH3 protein 1 | -1.250 | 7.41E-05 | 1448207_at |  |
| ICAM1 | intercellular adhesion molecule 1 | -1.249 | 1.87E-04 | 1424067_at |  |
| NEDD4L | neural precursor cell expressed, developmentally down-regulated 4-like | -1.248 | 6.45E-04 | 1423269_a_at | R |
| EIF3C | eukaryotic translation initiation factor 3, subunit C | -1.248 | 3.22E-04 | 1415859_at |  |
| Unknown | Unknown | -1.247 | 1.13E-04 | 1433924_at |  |
| S100A8 | S100 calcium binding protein A8 | -1.247 | 7.80E-05 | 1419394_s_at |  |
| UBE2E2 | ubiquitin-conjugating enzyme E2E 2 (UBC4/5 homolog, yeast) | -1.247 | 1.29E-04 | 1424358_at |  |
| FGL1 | fibrinogen-like 1 | -1.246 | 3.16E-04 | 1424599_at |  |
| NFYB | nuclear transcription factor Y, beta | -1.245 | 1.58E-04 | 1419267_at |  |
| CKS1B | CDC28 protein kinase regulatory subunit 1B | -1.243 | 7.60E-04 | 1416698_a_at |  |
| GPR98 | G protein-coupled receptor 98 | -1.242 | 7.39E-04 | 1425314_at |  |
| Unknown | Unknown | -1.241 | 5.43E-05 | 1443505_at |  |
| FERMT3 | fermitin family homolog 3 (Drosophila) | -1.240 | 1.58E-06 | 1433963_a_at |  |
| HSPA8 | heat shock 70kDa protein 8 | -1.240 | 1.56E-04 | 1431182_at |  |
| LAPTM5 | lysosomal protein transmembrane 5 | -1.240 | 2.11E-04 | 1436905_x_at |  |
| SREBF2 | sterol regulatory element binding transcription factor 2 | -1.240 | 6.23E-04 | 1426744_at |  |
| PLSCR1 | phospholipid scramblase 1 | -1.237 | 1.78E-04 | 1429527_a_at | R |
| ARMCX3 | armadillo repeat containing, X-linked 3 | -1.236 | 2.67E-04 | 1460359_at |  |
| FAM149A | family with sequence similarity 149, member A | -1.236 | 1.53E-04 | 1437950_at |  |
| DDX39 | DEAD (Asp-Glu-Ala-Asp) box polypeptide 39 | -1.235 | 3.04E-04 | 1451065_a_at |  |
| Unknown | Unknown | -1.234 | 1.39E-04 | 1437600_at |  |
| 9130219A07RIK | RIKEN cDNA 9130219A07 gene | -1.234 | 5.48E-05 | 1445457_at |  |
| REEP5 | receptor accessory protein 5 | -1.233 | 3.56E-04 | 1426376_at | R |
| EFNA1 | ephrin-A1 | -1.233 | 4.47E-04 | 1448510_at |  |
| GADD45A | growth arrest and DNA-damage-inducible, alpha | -1.233 | 2.04E-04 | 1449519_at |  |
| PRTN3 | proteinase 3 | -1.233 | 1.90E-04 | 1419669_at |  |
| GPD2 | glycerol-3-phosphate dehydrogenase 2 (mitochondrial) | -1.232 | 3.41E-04 | 1452741_s_at | R |
| BACE1 | beta-site APP-cleaving enzyme 1 | -1.232 | 2.76E-04 | 1455826_a_at |  |
| SERF1A | small EDRK-rich factor 1A (telomeric) | -1.232 | 1.99E-04 | 1434291_a_at |  |
| SFRS3 | splicing factor, arginine/serine-rich 3 | -1.232 | 3.10E-04 | 1416152_a_at |  |
| CYP51A1 | cytochrome P450, family 51, subfamily A, polypeptide 1 | -1.231 | 5.40E-05 | 1422534_at | R |
| SLC13A3 | solute carrier family 13 (sodium-dependent dicarboxylate transporter), member 3 | -1.230 | 5.59E-04 | 1416560_at | R |
| C330046E03 | hypothetical protein C330046E03 | -1.230 | 2.12E-04 | 1440144_x_at |  |
| EFEMP1 | EGF-containing fibulin-like extracellular matrix protein 1 | -1.230 | 2.42E-04 | 1427183_at |  |
| MMGT1 | membrane magnesium transporter 1 | -1.230 | 3.71E-04 | 1436706_at |  |
| CELSR1 | cadherin, EGF LAG seven-pass G-type receptor 1 (flamingo homolog, Drosophila) | -1.229 | 1.51E-04 | 1418925_at |  |
| COL3A1 | collagen, type III, alpha 1 | -1.229 | 3.38E-04 | 1427883_a_at |  |
| GM7969 | predicted gene 7969 | -1.229 | 6.76E-04 | 1455240_x_at |  |
| Unknown | Unknown | -1.228 | 6.85E-05 | 1447891_at |  |
| FSCN1 | fascin homolog 1, actin-bundling protein (Strongylocentrotus purpuratus) | -1.228 | 1.81E-04 | 1416514_a_at |  |
| ACNAT2 | acyl-coenzyme A amino acid N-acyltransferase 2 | -1.227 | 6.48E-04 | 1425150_at |  |
| ATP2B1 | ATPase, Ca++ transporting, plasma membrane 1 | -1.227 | 6.82E-04 | 1428936_at |  |
| Unknown | Unknown | -1.225 | 2.92E-04 | 1427820_at |  |
| ABCD2 | ATP-binding cassette, sub-family D (ALD), member 2 | -1.225 | 1.64E-04 | 1419748_at |  |
| SMYD1 | SET and MYND domain containing 1 | -1.225 | 1.46E-05 | 1441667_s_at |  |
| MVD | mevalonate (diphospho) decarboxylase | -1.224 | 3.70E-04 | 1448663_s_at |  |
| CUX1 | cut-like homeobox 1 | -1.223 | 3.66E-05 | 1441956_s_at |  |
| LSS | lanosterol synthase (2,3-oxidosqualene-lanosterol cyclase) | -1.223 | 2.03E-04 | 1426913_at |  |
| CAND1 | cullin-associated and neddylation-dissociated 1 | -1.222 | 1.27E-04 | 1455751_at |  |
| LY6D | lymphocyte antigen 6 complex, locus D | -1.222 | 4.17E-04 | 1416930_at |  |
| RPL5 | ribosomal protein L5 | -1.222 | 5.81E-04 | 1451077_at |  |
| DEFA24 | defensin, alpha, 24 | -1.221 | 9.70E-05 | 1450631_x_at |  |
| VASN | vasorin | -1.221 | 2.98E-04 | 1455812_x_at |  |
| HBA2 | hemoglobin, alpha 2 | -1.220 | 4.72E-04 | 1452757_s_at | R |
| SSR1 | signal sequence receptor, alpha | -1.220 | 3.62E-04 | 1448843_at | R |
| CSF2RB | colony stimulating factor 2 receptor, beta, low-affinity (granulocyte-macrophage) | -1.220 | 5.37E-04 | 1455660_at |  |
| NANS | N-acetylneuraminic acid synthase | -1.220 | 5.02E-04 | 1417774_at |  |
| SFRS1 | splicing factor, arginine/serine-rich 1 | -1.220 | 7.17E-04 | 1428100_at |  |
| PLSCR1 | phospholipid scramblase 1 | -1.219 | 6.92E-04 | 1453181_x_at | R |
| PFN2 | profilin 2 | -1.219 | 4.48E-04 | 1418209_a_at |  |
| Unknown | Unknown | -1.218 | 7.68E-04 | 1442531_at |  |
| CXADR | coxsackie virus and adenovirus receptor | -1.218 | 4.28E-04 | 1452391_at |  |
| LILRB4 | leukocyte immunoglobulin-like receptor, subfamily B (with TM and ITIM domains), member 4 | -1.216 | 6.17E-04 | 1420394_s_at |  |
| C4BP | complement component 4 binding protein | -1.215 | 2.64E-04 | 1418037_at |  |
| USP7 | ubiquitin specific peptidase 7 (herpes virus-associated) | -1.215 | 4.19E-04 | 1437118_at |  |
| CORO1A | coronin, actin binding protein, 1A | -1.214 | 7.50E-05 | 1455269_a_at |  |
| HIST1H2AE | histone cluster 1, H2ae | -1.214 | 9.71E-04 | 1438009_at |  |
| RNF14 | ring finger protein 14 | -1.213 | 4.25E-04 | 1431030_a_at |  |
| UBE2N | ubiquitin-conjugating enzyme E2N (UBC13 homolog, yeast) | -1.213 | 5.45E-04 | 1435384_at |  |
| RAB34 | RAB34, member RAS oncogene family | -1.212 | 7.80E-04 | 1416591_at |  |
| STXBP6 | syntaxin binding protein 6 (amisyn) | -1.212 | 7.59E-04 | 1435396_at |  |
| GBP6 | guanylate binding protein family, member 6 | -1.211 | 7.82E-04 | 1438676_at | R |
| ATG16L2 | ATG16 autophagy related 16-like 2 (S. cerevisiae) | -1.211 | 9.08E-04 | 1429396_at |  |
| WDR12 | WD repeat domain 12 | -1.211 | 3.97E-04 | 1417270_at |  |
| PPIL1 | peptidylprolyl isomerase (cyclophilin)-like 1 | -1.210 | 7.98E-04 | 1428892_at |  |
| ANKRD11 | ankyrin repeat domain 11 | -1.208 | 8.90E-04 | 1454863_at |  |
| LRPAP1 | low density lipoprotein receptor-related protein associated protein 1 | -1.208 | 9.11E-04 | 1452148_at |  |
| LOC440498 | heat shock factor binding protein 1-like | -1.207 | 4.92E-05 | 1444790_at |  |
| RNF170 | ring finger protein 170 | -1.207 | 9.74E-04 | 1437207_at |  |
| Unknown | Unknown | -1.206 | 5.84E-04 | 1440325_at |  |
| Unknown | Unknown | -1.206 | 4.59E-04 | 1456868_at |  |
| MAGEH1 | melanoma antigen family H, 1 | -1.206 | 1.64E-04 | 1422498_at |  |
| PLA2G6 | phospholipase A2, group VI (cytosolic, calcium-independent) | -1.206 | 8.76E-04 | 1431278_s_at |  |
| SERPINA3M | serine (or cysteine) peptidase inhibitor, clade A, member 3M | -1.206 | 6.12E-04 | 1421921_at |  |
| ZEB2 | zinc finger E-box binding homeobox 2 | -1.206 | 8.97E-04 | 1422748_at |  |
| GUSB | glucuronidase, beta | -1.205 | 6.14E-04 | 1430332_a_at |  |
| IGDCC4 | immunoglobulin superfamily, DCC subclass, member 4 | -1.205 | 5.82E-04 | 1416473_a_at |  |
| INTS2 | integrator complex subunit 2 | -1.205 | 1.19E-04 | 1436247_at |  |
| LILRA6 | leukocyte immunoglobulin-like receptor, subfamily A (with TM domain), member 6 | -1.204 | 1.25E-04 | 1452487_x_at | R |
| TGM1 | transglutaminase 1 (K polypeptide epidermal type I, protein-glutamine-gamma-glutamyltransferase) | -1.203 | 9.52E-04 | 1451416_a_at |  |
| VIM | vimentin | -1.201 | 5.01E-04 | 1456292_a_at |  |
| ACER3 | alkaline ceramidase 3 | -1.194 | 5.20E-04 | 1459771_x_at |  |
| CXCL14 | chemokine (C-X-C motif) ligand 14 | -1.193 | 3.63E-04 | 1418457_at |  |
| HAMP | hepcidin antimicrobial peptide | -1.189 | 1.80E-05 | 1419197_x_at | R |
| ZC3H12A | zinc finger CCCH-type containing 12A | -1.184 | 6.84E-04 | 1427348_at |  |
| CCDC25 | coiled-coil domain containing 25 | -1.180 | 9.89E-04 | 1451799_at |  |
| SCD | stearoyl-CoA desaturase (delta-9-desaturase) | -1.179 | 1.24E-05 | 1415964_at | R |
| Unknown | Unknown | -1.178 | 3.59E-04 | 1440461_at |  |
| CD40 | CD40 molecule, TNF receptor superfamily member 5 | -1.173 | 4.44E-04 | 1439221_s_at |  |
| KRT28 | keratin 28 | -1.166 | 2.18E-04 | 1458354_x_at |  |
| RP2 | retinitis pigmentosa 2 (X-linked recessive) | -1.166 | 2.25E-04 | 1419587_s_at |  |
| PTAFR | platelet-activating factor receptor | -1.160 | 8.88E-04 | 1427871_at |  |
| HAMP | hepcidin antimicrobial peptide | -1.145 | 4.65E-04 | 1419196_at | R |
| GMDS | GDP-mannose 4,6-dehydratase | -1.139 | 2.07E-06 | 1439428_x_at | R |
| ITIH4 | inter-alpha (globulin) inhibitor H4 (plasma Kallikrein-sensitive glycoprotein) | -1.137 | 2.01E-04 | 1431808_a_at |  |
| 9330109K16RIK | RIKEN cDNA 9330109K16 gene | 1.109 | 4.65E-04 | 1457747_at |  |
| LUZP2 | leucine zipper protein 2 | 1.127 | 3.61E-04 | 1441913_at |  |
| SLBP | stem-loop binding protein | 1.145 | 8.58E-04 | 1437803_at |  |
| Unknown | Unknown | 1.149 | 8.64E-04 | 1458301_x_at |  |
| HAPLN1 | hyaluronan and proteoglycan link protein 1 | 1.153 | 2.97E-04 | 1426295_at | R |
| CPS1 | carbamoyl-phosphate synthetase 1, mitochondrial | 1.161 | 2.75E-05 | 1455540_at |  |
| 9630041G16RIK | RIKEN cDNA 9630041G16 gene | 1.166 | 2.44E-04 | 1445713_at |  |
| CYP1B1 | cytochrome P450, family 1, subfamily B, polypeptide 1 | 1.170 | 1.09E-04 | 1416613_at |  |
| Unknown | Unknown | 1.173 | 9.13E-04 | 1441784_at |  |
| GNMT | glycine N-methyltransferase | 1.174 | 6.81E-06 | 1417422_at |  |
| A930035E12RIK | RIKEN cDNA A930035E12 gene | 1.178 | 4.68E-04 | 1429906_at |  |
| SLC38A4 | solute carrier family 38, member 4 | 1.178 | 2.14E-04 | 1448889_at |  |
| TMCO5A | transmembrane and coiled-coil domains 5A | 1.181 | 7.56E-04 | 1459831_s_at |  |
| CYP1A2 | cytochrome P450, family 1, subfamily A, polypeptide 2 | 1.182 | 1.59E-04 | 1450715_at |  |
| 9130022000000000 | hypothetical 9130022E09 | 1.183 | 5.90E-04 | 1429344_at |  |
| PAIP1 | poly(A) binding protein interacting protein 1 | 1.183 | 8.55E-04 | 1441955_s_at |  |
| B630019K06RIK | RIKEN cDNA B630019K06 gene | 1.186 | 2.77E-04 | 1447618_at |  |
| Unknown | Unknown | 1.187 | 6.43E-04 | 1446132_at |  |
| TCSTV3 | 2-cell-stage, variable group, member 3 | 1.191 | 8.09E-04 | 1451852_at |  |
| NCOA1 | nuclear receptor coactivator 1 | 1.193 | 5.31E-04 | 1443397_at |  |
| FMO2 | flavin containing monooxygenase 2 (non-functional) | 1.195 | 9.80E-04 | 1454005_at |  |
| CPSF3 | cleavage and polyadenylation specific factor 3, 73kDa | 1.198 | 3.74E-04 | 1437328_x_at |  |
| LOC624112 | hypothetical protein LOC624112 | 1.199 | 1.29E-04 | 1440815_x_at |  |
| Unknown | Unknown | 1.200 | 9.19E-04 | 1446704_at |  |
| TAT | tyrosine aminotransferase | 1.200 | 3.07E-04 | 1451557_at |  |
| Unknown | Unknown | 1.201 | 3.56E-05 | 1445409_at |  |
| MIPOL1 | mirror-image polydactyly 1 | 1.201 | 5.16E-04 | 1435671_at |  |
| Unknown | Unknown | 1.202 | 8.39E-04 | 1446241_at |  |
| Unknown | Unknown | 1.202 | 7.12E-04 | 1458754_at |  |
| ADAM6B | a disintegrin and metallopeptidase domain 6B | 1.202 | 8.33E-04 | 1436129_at | R |
| CCDC39 | coiled-coil domain containing 39 | 1.202 | 4.25E-05 | 1440504_at |  |
| OPRK1 | opioid receptor, kappa 1 | 1.203 | 8.66E-04 | 1446394_at |  |
| 4930468A15RIK | RIKEN cDNA 4930468A15 gene | 1.204 | 7.24E-04 | 1431639_at |  |
| TSSK4 | testis-specific serine kinase 4 | 1.204 | 8.99E-04 | 1443810_at |  |
| GM6150 | predicted gene 6150 | 1.207 | 5.19E-04 | 1439338_at |  |
| Unknown | Unknown | 1.208 | 7.58E-04 | 1456254_at |  |
| 5430402O13RIK | RIKEN cDNA 5430402O13 gene | 1.208 | 3.10E-04 | 1454463_at |  |
| TET1 | tet oncogene 1 | 1.208 | 5.31E-04 | 1455425_at |  |
| APOD | apolipoprotein D | 1.209 | 6.73E-04 | 1444564_at |  |
| Unknown | Unknown | 1.210 | 4.49E-04 | 1419830_at |  |
| 5830468K08RIK | RIKEN cDNA 5830468K08 gene | 1.210 | 6.30E-04 | 1432588_at |  |
| Unknown | Unknown | 1.211 | 3.78E-04 | 1440750_at |  |
| TMEM47 | transmembrane protein 47 | 1.211 | 3.91E-04 | 1449885_at |  |
| A930017M01RIK | RIKEN cDNA A930017M01 gene | 1.212 | 6.78E-04 | 1437076_at |  |
| COX7C | cytochrome c oxidase subunit VIIc | 1.212 | 5.91E-04 | 1459885_s_at |  |
| LONRF2 | LON peptidase N-terminal domain and ring finger 2 | 1.213 | 6.76E-04 | 1429965_at |  |
| ZNF808 | zinc finger protein 808 | 1.213 | 8.87E-04 | 1449972_s_at |  |
| NEB | nebulin | 1.215 | 6.84E-04 | 1435355_at |  |
| Unknown | Unknown | 1.216 | 8.18E-04 | 1442545_at |  |
| Unknown | Unknown | 1.216 | 4.29E-04 | 1444522_at |  |
| ALDH1L1 | aldehyde dehydrogenase 1 family, member L1 | 1.216 | 1.68E-06 | 1424400_a_at |  |
| SPARCL1 | SPARC-like 1 (hevin) | 1.216 | 8.50E-04 | 1416114_at |  |
| ARNTL2 | aryl hydrocarbon receptor nuclear translocator-like 2 | 1.218 | 8.96E-05 | 1429688_at |  |
| GM11944 | predicted gene 11944 | 1.218 | 5.28E-04 | 1446846_at |  |
| SULT1A1 | sulfotransferase family, cytosolic, 1A, phenol-preferring, member 1 | 1.218 | 8.64E-05 | 1427345_a_at |  |
| PLEKHH1 | pleckstrin homology domain containing, family H (with MyTH4 domain) member 1 | 1.219 | 3.50E-04 | 1452517_at |  |
| TPT1 | tumor protein, translationally-controlled 1 | 1.219 | 3.84E-04 | 1416643_at |  |
| BTNL2 | butyrophilin-like 2 (MHC class II associated) | 1.222 | 6.39E-04 | 1442199_at |  |
| Unknown | Unknown | 1.224 | 8.13E-04 | 1445574_at |  |
| ASL | argininosuccinate lyase | 1.224 | 4.36E-04 | 1448350_at |  |
| IGFBP2 | insulin-like growth factor binding protein 2, 36kDa | 1.226 | 1.52E-04 | 1454159_a_at |  |
| TRIB3 | tribbles homolog 3 (Drosophila) | 1.226 | 5.26E-04 | 1426065_a_at |  |
| APCDD1 | adenomatosis polyposis coli down-regulated 1 | 1.228 | 4.54E-04 | 1443639_at |  |
| C13ORF15 | chromosome 13 open reading frame 15 | 1.228 | 3.51E-04 | 1438511_a_at |  |
| RSAD2 | radical S-adenosyl methionine domain containing 2 | 1.228 | 2.29E-04 | 1421009_at |  |
| Unknown | Unknown | 1.229 | 6.08E-04 | 1443780_at |  |
| MSI2 | musashi homolog 2 (Drosophila) | 1.231 | 7.05E-04 | 1435521_at |  |
| INMT | indolethylamine N-methyltransferase | 1.232 | 3.30E-05 | 1418697_at |  |
| RASSF5 | Ras association (RalGDS/AF-6) domain family member 5 | 1.233 | 1.93E-04 | 1422637_at |  |
| KYNU | kynureninase (L-kynurenine hydrolase) | 1.234 | 2.04E-04 | 1451903_at |  |
| GPT | glutamic-pyruvate transaminase (alanine aminotransferase) | 1.235 | 2.83E-04 | 1426502_s_at |  |
| MAP3K7IP1 | mitogen-activated protein kinase kinase kinase 7 interacting protein 1 | 1.236 | 1.68E-04 | 1447692_x_at |  |
| CEP110 | centrosomal protein 110kDa | 1.237 | 9.23E-04 | 1435779_at |  |
| GABARAPL1 | GABA(A) receptor-associated protein like 1 | 1.238 | 2.26E-05 | 1416419_s_at | R |
| PDE4B | phosphodiesterase 4B, cAMP-specific (phosphodiesterase E4 dunce homolog, Drosophila) | 1.238 | 2.39E-05 | 1447718_at |  |
| A330068G13RIK | RIKEN cDNA A330068G13 gene | 1.239 | 4.83E-05 | 1441491_at |  |
| HIVEP2 | human immunodeficiency virus type I enhancer binding protein 2 | 1.239 | 1.79E-04 | 1422018_at |  |
| ACADL | acyl-Coenzyme A dehydrogenase, long chain | 1.240 | 2.59E-05 | 1448988_at |  |
| Unknown | Unknown | 1.241 | 1.46E-04 | 1436633_at |  |
| Unknown | Unknown | 1.242 | 1.36E-04 | 1441014_at |  |
| SHH | sonic hedgehog homolog (Drosophila) | 1.242 | 7.12E-04 | 1436869_at |  |
| C14ORF106 | chromosome 14 open reading frame 106 | 1.245 | 1.29E-04 | 1434767_at |  |
| AVL9 | AVL9 homolog (S. cerevisiase) | 1.246 | 2.81E-04 | 1457023_at |  |
| PVR | poliovirus receptor | 1.248 | 6.23E-04 | 1423904_a_at |  |
| CALM3 | calmodulin 3 (phosphorylase kinase, delta) | 1.249 | 7.53E-05 | 1438825_at |  |
| SOX4 | SRY (sex determining region Y)-box 4 | 1.252 | 3.94E-04 | 1443778_at |  |
| SNORD22 | small nucleolar RNA, C/D box 22 | 1.253 | 8.24E-04 | 1439399_a_at | R |
| ZEB1 | zinc finger E-box binding homeobox 1 | 1.253 | 2.04E-04 | 1420243_at |  |
| TMEM184C | transmembrane protein 184C | 1.256 | 1.15E-04 | 1447610_at |  |
| FUBP1 | far upstream element (FUSE) binding protein 1 | 1.258 | 6.58E-05 | 1433482_a_at |  |
| PIGO | phosphatidylinositol glycan anchor biosynthesis, class O | 1.259 | 7.11E-04 | 1437142_a_at |  |
| Unknown | Unknown | 1.260 | 3.32E-06 | 1442879_at |  |
| IFIT1L | interferon-induced protein with tetratricopeptide repeats 1-like | 1.260 | 5.91E-04 | 1450783_at |  |
| SLC16A5 | solute carrier family 16, member 5 (monocarboxylic acid transporter 6) | 1.260 | 4.74E-05 | 1434473_at |  |
| TFRC | transferrin receptor (p90, CD71) | 1.260 | 2.50E-04 | 1452661_at |  |
| TMEM59 | transmembrane protein 59 | 1.260 | 8.09E-05 | 1450046_at |  |
| HAPLN1 | hyaluronan and proteoglycan link protein 1 | 1.261 | 5.07E-06 | 1421633_a_at | R |
| DUS4L | dihydrouridine synthase 4-like (S. cerevisiae) | 1.261 | 3.29E-05 | 1441006_at |  |
| Unknown | Unknown | 1.263 | 8.41E-06 | 1420292_x_at |  |
| FAM134B | family with sequence similarity 134, member B | 1.263 | 3.88E-04 | 1424683_at |  |
| WDR18 | WD repeat domain 18 | 1.266 | 8.26E-05 | 1443758_at |  |
| RNF114 | ring finger protein 114 | 1.267 | 6.47E-04 | 1437949_x_at |  |
| ADAM6B | a disintegrin and metallopeptidase domain 6B | 1.269 | 8.15E-05 | 1436130_s_at | R |
| GSTT2 | glutathione S-transferase theta 2 | 1.272 | 6.84E-04 | 1417883_at |  |
| Unknown | Unknown | 1.273 | 2.06E-05 | 1459072_at |  |
| CYP2B6 | cytochrome P450, family 2, subfamily B, polypeptide 6 | 1.274 | 7.85E-05 | 1422257_s_at | R |
| GABARAPL1 | GABA(A) receptor-associated protein like 1 | 1.274 | 2.03E-04 | 1416418_at | R |
| Unknown | Unknown | 1.279 | 3.91E-08 | 1438926_at |  |
| SNORD22 | small nucleolar RNA, C/D box 22 | 1.283 | 3.08E-05 | 1433675_at | R |
| ANK3 | ankyrin 3, node of Ranvier (ankyrin G) | 1.283 | 2.97E-05 | 1452124_at |  |
| Unknown | Unknown | 1.287 | 2.81E-07 | 1420212_at |  |
| Unknown | Unknown | 1.288 | 8.48E-06 | 1420291_at |  |
| KIFC2 | kinesin family member C2 | 1.288 | 2.66E-04 | 1421312_a_at |  |
| ASS1 | argininosuccinate synthetase 1 | 1.291 | 1.07E-13 | 1416239_at |  |
| KCND2 | potassium voltage-gated channel, Shal-related subfamily, member 2 | 1.291 | 1.55E-04 | 1447764_at |  |
| LOC647310 | similar to testis expressed gene 22 | 1.291 | 3.41E-06 | 1429548_at |  |
| USP2 | ubiquitin specific peptidase 2 | 1.291 | 2.22E-04 | 1417168_a_at |  |
| CLEC2D | C-type lectin domain family 2, member D | 1.294 | 1.46E-05 | 1451438_s_at | R |
| CTH | cystathionase (cystathionine gamma-lyase) | 1.294 | 7.39E-06 | 1426243_at |  |
| PIK3CD | phosphoinositide-3-kinase, catalytic, delta polypeptide | 1.294 | 6.71E-06 | 1458321_at |  |
| RP5-1022P6.2 | hypothetical protein KIAA1434 | 1.294 | 2.20E-05 | 1437953_at |  |
| CYP4A14 | cytochrome P450, family 4, subfamily a, polypeptide 14 | 1.300 | 8.67E-05 | 1423257_at |  |
| SLCO1A2 | solute carrier organic anion transporter family, member 1A2 | 1.304 | 4.06E-05 | 1420405_at |  |
| SLC7A2 | solute carrier family 7 (cationic amino acid transporter, y+ system), member 2 | 1.305 | 2.66E-05 | 1450703_at | R |
| ALDH1B1 | aldehyde dehydrogenase 1 family, member B1 | 1.306 | 1.66E-05 | 1451260_at |  |
| ADORA1 | adenosine A1 receptor | 1.308 | 1.17E-06 | 1435495_at |  |
| AFMID | arylformamidase | 1.312 | 9.63E-05 | 1452944_at |  |
| IVD | isovaleryl Coenzyme A dehydrogenase | 1.314 | 1.66E-04 | 1449001_at |  |
| DBP | D site of albumin promoter (albumin D-box) binding protein | 1.317 | 4.81E-05 | 1418174_at |  |
| TK1 | thymidine kinase 1, soluble | 1.321 | 4.40E-05 | 1416258_at |  |
| AGXT | alanine-glyoxylate aminotransferase | 1.324 | 1.11E-06 | 1418833_at |  |
| Unknown | Unknown | 1.328 | 4.30E-05 | 1447699_at |  |
| RNASE2 | ribonuclease, RNase A family, 2 (liver, eosinophil-derived neurotoxin) | 1.328 | 1.94E-07 | 1425295_at |  |
| GCNT2 | glucosaminyl (N-acetyl) transferase 2, I-branching enzyme (I blood group) | 1.329 | 8.98E-07 | 1437607_at |  |
| GLS2 | glutaminase 2 (liver, mitochondrial) | 1.332 | 2.06E-06 | 1435245_at |  |
| OAT | ornithine aminotransferase (gyrate atrophy) | 1.333 | 2.97E-10 | 1416452_at |  |
| 1500017E21RIK | RIKEN cDNA 1500017E21 gene | 1.334 | 1.68E-05 | 1438596_at |  |
| CYP2B9 | cytochrome P450, family 2, subfamily b, polypeptide 9 | 1.339 | 1.78E-05 | 1419590_at |  |
| DCXR | dicarbonyl/L-xylulose reductase | 1.339 | 9.93E-06 | 1419456_at |  |
| SLC43A1 | solute carrier family 43, member 1 | 1.339 | 3.01E-07 | 1453255_at |  |
| CYP2B6 | cytochrome P450, family 2, subfamily B, polypeptide 6 | 1.341 | 1.04E-06 | 1425645_s_at | R |
| CD163L1 | CD163 molecule-like 1 | 1.345 | 6.85E-06 | 1440808_x_at |  |
| Unknown | Unknown | 1.351 | 3.97E-08 | 1447698_x_at |  |
| GSTT3 | glutathione S-transferase, theta 3 | 1.351 | 1.84E-05 | 1423891_at |  |
| UPP2 | uridine phosphorylase 2 | 1.355 | 4.09E-06 | 1460059_at | R |
| ANKHD1 | ankyrin repeat and KH domain containing 1 | 1.357 | 3.11E-06 | 1453023_at |  |
| CYP2A13 | cytochrome P450, family 2, subfamily A, polypeptide 13 | 1.358 | 1.13E-09 | 1422230_s_at |  |
| MT1E | metallothionein 1E | 1.365 | 4.05E-06 | 1428942_at |  |
| NNMT | nicotinamide N-methyltransferase | 1.374 | 8.92E-07 | 1432517_a_at |  |
| CREB1 | cAMP responsive element binding protein 1 | 1.381 | 1.28E-08 | 1421583_at |  |
| CORO1C | coronin, actin binding protein, 1C | 1.396 | 2.01E-06 | 1437721_at |  |
| SDS | serine dehydratase | 1.404 | 5.39E-10 | 1424744_at |  |
| Unknown | Unknown | 1.406 | 3.04E-08 | 1440506_at |  |
| SLC7A2 | solute carrier family 7 (cationic amino acid transporter, y+ system), member 2 | 1.418 | 1.30E-09 | 1436555_at | R |
| UPP2 | uridine phosphorylase 2 | 1.438 | 3.92E-10 | 1424969_s_at | R |
| BTN1A1 | butyrophilin, subfamily 1, member A1 | 1.459 | 7.72E-09 | 1425754_a_at |  |
| SLC7A2 | solute carrier family 7 (cationic amino acid transporter, y+ system), member 2 | 1.473 | 1.31E-08 | 1422648_at | R |
| ABHD5 | abhydrolase domain containing 5 | 1.479 | 2.56E-08 | 1447548_at |  |
| GOT1 | glutamic-oxaloacetic transaminase 1, soluble (aspartate aminotransferase 1) | 1.505 | 7.12E-11 | 1450970_at |  |
| SLC7A2 | solute carrier family 7 (cationic amino acid transporter, y+ system), member 2 | 1.546 | 7.19E-11 | 1426008_a_at | R |
| UPP2 | uridine phosphorylase 2 | 1.552 | 5.07E-11 | 1451548_at | R |
| GSTA5 | glutathione S-transferase alpha 5 | 1.568 | 1.52E-12 | 1421041_s_at | R |
| GSTA5 | glutathione S-transferase alpha 5 | 1.621 | 6.55E-13 | 1421040_a_at | R |
| MT1F | metallothionein 1F | 1.631 | 1.79E-13 | 1422557_s_at |  |
| CYP7A1 | cytochrome P450, family 7, subfamily A, polypeptide 1 | 1.652 | 3.33E-14 | 1438743_at | R |
| AGXT2L1 | alanine-glyoxylate aminotransferase 2-like 1 | 1.675 | 3.09E-14 | 1452975_at | R |
| CLEC2D | C-type lectin domain family 2, member D | 1.685 | 2.59E-18 | 1431240_at | R |
| Unknown | Unknown | 1.693 | 1.96E-19 | 1441430_at |  |
| CYP7A1 | cytochrome P450, family 7, subfamily A, polypeptide 1 | 1.769 | 4.68E-16 | 1422100_at | R |
| AGXT2L1 | alanine-glyoxylate aminotransferase 2-like 1 | 1.860 | 9.77E-22 | 1431406_at | R |
| CLEC2D | C-type lectin domain family 2, member D | 1.932 | 1.30E-29 | 1424673_at | R |

All differentially expressed transcripts (p<0.001) resulting from a comparison of H-P/C-HF with L-P/C-HF mice after 2-d of feeding are listed. The significance of differences was estimated by a moderated ANOVA as described in the Material and Method section. The fold change with a negative and a positive value indicates down-regulation and up-regulation in the H-P/C-HF group, respectively. An Affymetrix probeset ID (Mouse 430 2.0) is provided for each gene. R in Notes represents a replicated detection with an alternative probeset for a given gene on the microarray.
