# Supplementary material for: Consequences of Exchanging Carbohydrates for Proteins in the Cholesterol Metabolism of Mice Fed a High-fat Diet
Source: PLoS One. 2012 Nov 6;7(11):e49058. doi: 10.1371/journal.pone.0049058 (PMC3490911; doi:10.1371/journal.pone.0049058)
Supplement: Table S3 — Transcription factor analysis of global gene expressions at 2-d after H-P/C-HF feeding. (DOC) [file pone.0049058.s006.doc]

**Table S3. Transcription factor analysis of global gene expressions at 2-d after H-P/C-HF feeding**

| Upstream Regulator | Fold Change | Molecule Type | Predicted Activation State | p-value of overlap | Target molecules in dataset |
| --- | --- | --- | --- | --- | --- |
| AHR |  | ligand-dependent nuclear receptor | Activated | 1.29E-10 | A2M, ACOX1, ALDH1B1, COL3A1, CYP1A2, CYP1B1, CYP2A6 (includes others), CYP2B6, EFEMP1, FABP4 |
| NR1I3 |  | ligand-dependent nuclear receptor | Activated | 1.02E-07 | ACOX1, APCS, CYP1A2, CYP2A6 (includes others), Cyp2b13/Cyp2b9, CYP2B6, CYP7A1, EPHX1, GSTA5, ICAM1 |
| SREBF2 | -1.240 | transcription regulator | Inhibited | 3.62E-16 | ACLY, CYP51A1, FABP5, FASN, FDFT1, FDPS, HBA1/HBA2, HMGCS1, IDI1, LSS |
| SREBF1 (includes EG:176574) |  | transcription regulator | Inhibited | 8.87E-14 | ABCD2, ACLY, CSAD, CYP51A1, CYP7A1, FABP4, FABP5, FASN, FDFT1, FDPS |
| CEBPB (includes EG:1051) |  | transcription regulator | Inhibited | 1.39E-07 | APCS, ASNS, CP, CTSC, CXCL2, CYP2A6 (includes others), FABP4, FOS, G6PC, GADD45A |
