# Supplementary material for: Consequences of Exchanging Carbohydrates for Proteins in the Cholesterol Metabolism of Mice Fed a High-fat Diet
Source: PLoS One. 2012 Nov 6;7(11):e49058. doi: 10.1371/journal.pone.0049058 (PMC3490911; doi:10.1371/journal.pone.0049058)
Supplement: Table S4 — List of regulated transcripts after H-P/C-HF feeding for 4 wks. All differentially expressed transcripts (p<0.001) resulting from a comparison of H-P/C-HF with L-P/C-HF mice after 4-wk of feeding are listed. The significance of differences was estimated by a moderated ANOVA as described in the Material and Method section. The fold change with a negative and a positive value indicates down-regulation and up-regulation in the H-P/C-HF group, respectively. An Affymetrix probeset ID (Mouse 430 2.0) is provided for each gene. R in Notes represents a replicated detection with a different probeset for a given gene on the microarray. (DOC) [file pone.0049058.s007.doc]

Table S4. List of regulated transcripts after H-P/C-HF feeding for 4 wks

| ***Symbol*** | ***Entrez Gene Name*** | ***Fold Change compared to L-P/C-HF*** | ***p-value*** | ***ProbeSet ID*** | ***Notes*** |
| --- | --- | --- | --- | --- | --- |
| MARCO | macrophage receptor with collagenous structure | -1.876 | 2.18E-34 | 1449498_at | R |
| SLC22A7 | solute carrier family 22 (organic anion transporter), member 7 | -1.661 | 1.60E-19 | 1451460_a_at |  |
| CXCL13 | chemokine (C-X-C motif) ligand 13 | -1.640 | 2.21E-21 | 1417851_at |  |
| BACH2 | BTB and CNC homology 1, basic leucine zipper transcription factor 2 | -1.599 | 3.74E-19 | 1436515_at | R |
| CFD | complement factor D (adipsin) | -1.517 | 4.09E-16 | 1417867_at |  |
| MARCO | macrophage receptor with collagenous structure | -1.516 | 1.89E-15 | 1458297_s_at | R |
| AQP4 | aquaporin 4 | -1.508 | 1.60E-15 | 1434449_at | R |
| BACH2 | BTB and CNC homology 1, basic leucine zipper transcription factor 2 | -1.465 | 7.44E-12 | 1437667_a_at | R |
| TSC22D1 | TSC22 domain family, member 1 | -1.458 | 2.54E-11 | 1425742_a_at | R |
| SERPINA12 | serpin peptidase inhibitor, clade A (alpha-1 antiproteinase, antitrypsin), member 12 | -1.454 | 1.24E-16 | 1421092_at |  |
| RAD51L1 | RAD51-like 1 (S. cerevisiae) | -1.449 | 1.46E-12 | 1421430_at |  |
| OBP2B | odorant binding protein 2B | -1.441 | 4.46E-12 | 1428022_at |  |
| TSC22D1 | TSC22 domain family, member 1 | -1.436 | 1.48E-10 | 1454758_a_at | R |
| GNA14 | guanine nucleotide binding protein (G protein), alpha 14 | -1.431 | 9.45E-12 | 1449848_at | R |
| SAA2 | serum amyloid A2 | -1.431 | 1.10E-10 | 1419075_s_at | R |
| CPEB2 | cytoplasmic polyadenylation element binding protein 2 | -1.415 | 4.58E-11 | 1443017_at | R |
| DERL3 | Der1-like domain family, member 3 | -1.404 | 2.62E-09 | 1453677_a_at |  |
| GNA14 | guanine nucleotide binding protein (G protein), alpha 14 | -1.401 | 3.48E-12 | 1420385_at | R |
| DUSP6 | dual specificity phosphatase 6 | -1.400 | 1.77E-09 | 1415834_at |  |
| LYZ | lysozyme (renal amyloidosis) | -1.398 | 3.45E-09 | 1436996_x_at | R |
| Unknown | Unknown | -1.392 | 2.65E-09 | 1443147_at |  |
| AQP4 | aquaporin 4 | -1.375 | 5.19E-10 | 1425382_a_at | R |
| IL2RG | interleukin 2 receptor, gamma (severe combined immunodeficiency) | -1.373 | 1.12E-09 | 1416295_a_at |  |
| SAA2 | serum amyloid A2 | -1.370 | 3.58E-08 | 1450788_at | R |
| TSC22D1 | TSC22 domain family, member 1 | -1.368 | 1.04E-08 | 1454971_x_at | R |
| LYZ | lysozyme (renal amyloidosis) | -1.367 | 4.65E-08 | 1439426_x_at | R |
| RARRES1 | retinoic acid receptor responder (tazarotene induced) 1 | -1.366 | 4.12E-08 | 1438055_at |  |
| DYNLL1 | dynein, light chain, LC8-type 1 | -1.360 | 4.57E-09 | 1417339_a_at | R |
| LYZ | lysozyme (renal amyloidosis) | -1.352 | 5.96E-08 | 1423547_at | R |
| MB | myoglobin | -1.350 | 9.25E-09 | 1451203_at |  |
| 2210023G05RIK | RIKEN cDNA 2210023G05 gene | -1.348 | 1.50E-07 | 1424968_at |  |
| NR0B2 | nuclear receptor subfamily 0, group B, member 2 | -1.345 | 9.36E-08 | 1449854_at |  |
| CCND1 | cyclin D1 | -1.343 | 5.04E-08 | 1417420_at | R |
| GNA14 | guanine nucleotide binding protein (G protein), alpha 14 | -1.342 | 1.43E-09 | 1447791_s_at | R |
| CA3 | carbonic anhydrase III, muscle specific | -1.341 | 3.09E-07 | 1430584_s_at | R |
| DYNLL1 | dynein, light chain, LC8-type 1 | -1.340 | 1.02E-07 | 1456125_a_at | R |
| LCN2 | lipocalin 2 | -1.338 | 5.92E-08 | 1427747_a_at |  |
| AIF1 | allograft inflammatory factor 1 | -1.333 | 1.19E-07 | 1418204_s_at |  |
| SAA2 | serum amyloid A2 | -1.333 | 1.39E-07 | 1449326_x_at | R |
| GM11428 | predicted gene 11428 | -1.329 | 1.45E-07 | 1436530_at |  |
| CA3 | carbonic anhydrase III, muscle specific | -1.325 | 6.26E-07 | 1453588_at | R |
| PPP1R3C | protein phosphatase 1, regulatory (inhibitor) subunit 3C | -1.325 | 4.29E-07 | 1433691_at | R |
| BMYC | brain expressed myelocytomatosis oncogene | -1.322 | 6.38E-07 | 1428669_at |  |
| HSD17B6 | hydroxysteroid (17-beta) dehydrogenase 6 homolog (mouse) | -1.319 | 3.16E-08 | 1449385_at |  |
| ERO1LB | ERO1-like beta (S. cerevisiae) | -1.316 | 3.26E-06 | 1425705_a_at | R |
| INSIG2 | insulin induced gene 2 | -1.313 | 7.68E-07 | 1417981_at | R |
| BCL6 | B-cell CLL/lymphoma 6 | -1.312 | 3.73E-07 | 1421818_at | R |
| LILRA6 | leukocyte immunoglobulin-like receptor, subfamily A (with TM domain), member 6 | -1.312 | 8.69E-08 | 1420464_s_at | R |
| MYH1 | myosin, heavy chain 1, skeletal muscle, adult | -1.309 | 6.24E-14 | 1427868_x_at | R |
| EMR1 | egf-like module containing, mucin-like, hormone receptor-like 1 | -1.306 | 1.78E-06 | 1451161_a_at |  |
| PPP1R3C | protein phosphatase 1, regulatory (inhibitor) subunit 3C | -1.306 | 3.54E-06 | 1425631_at | R |
| ERO1LB | ERO1-like beta (S. cerevisiae) | -1.302 | 1.29E-06 | 1434714_at | R |
| CFP | complement factor properdin | -1.300 | 2.75E-06 | 1452279_at |  |
| CLEC4A3 | C-type lectin domain family 4, member a3 | -1.300 | 8.23E-07 | 1429954_at |  |
| CPEB2 | cytoplasmic polyadenylation element binding protein 2 | -1.300 | 3.77E-06 | 1458518_at | R |
| ARRDC2 | arrestin domain containing 2 | -1.298 | 3.19E-07 | 1428352_at |  |
| OMD | osteomodulin | -1.292 | 1.04E-06 | 1418745_at |  |
| GPR98 | G protein-coupled receptor 98 | -1.291 | 2.39E-06 | 1425314_at |  |
| TNNT3 | troponin T type 3 (skeletal, fast) | -1.289 | 1.78E-07 | 1450118_a_at |  |
| COL27A1 | collagen, type XXVII, alpha 1 | -1.288 | 6.08E-06 | 1429549_at | R |
| CD5L | CD5 molecule-like | -1.286 | 8.15E-06 | 1449193_at |  |
| ABCG5 | ATP-binding cassette, sub-family G (WHITE), member 5 | -1.283 | 1.89E-06 | 1419393_at |  |
| C1QB | complement component 1, q subcomponent, B chain | -1.282 | 1.39E-05 | 1417063_at | R |
| EGR1 | early growth response 1 | -1.278 | 6.15E-06 | 1417065_at |  |
| MYH1 | myosin, heavy chain 1, skeletal muscle, adult | -1.278 | 7.95E-12 | 1427520_a_at | R |
| EMR4P | egf-like module containing, mucin-like, hormone receptor-like 4 pseudogene | -1.277 | 6.83E-06 | 1424998_at | R |
| ID2 | inhibitor of DNA binding 2, dominant negative helix-loop-helix protein | -1.277 | 1.89E-06 | 1453596_at | R |
| TREML4 | triggering receptor expressed on myeloid cells-like 4 | -1.277 | 1.81E-06 | 1460014_at |  |
| SLC25A25 | solute carrier family 25 (mitochondrial carrier; phosphate carrier), member 25 | -1.275 | 1.37E-05 | 1424735_at |  |
| TIMD4 | T-cell immunoglobulin and mucin domain containing 4 | -1.275 | 4.36E-06 | 1455318_at |  |
| G0S2 | G0/G1switch 2 | -1.274 | 1.80E-06 | 1448700_at |  |
| GPD2 | glycerol-3-phosphate dehydrogenase 2 (mitochondrial) | -1.273 | 3.74E-06 | 1417434_at | R |
| RNASE2 | ribonuclease, RNase A family, 2 (liver, eosinophil-derived neurotoxin) | -1.271 | 1.08E-06 | 1425295_at |  |
| 9030619P08RIK | RIKEN cDNA 9030619P08 gene | -1.268 | 6.46E-06 | 1443889_at |  |
| C1QA | complement component 1, q subcomponent, A chain | -1.264 | 3.67E-05 | 1417381_at |  |
| INSIG2 | insulin induced gene 2 | -1.263 | 2.83E-05 | 1417980_a_at | R |
| GPD2 | glycerol-3-phosphate dehydrogenase 2 (mitochondrial) | -1.262 | 4.39E-05 | 1428323_at | R |
| REEP5 | receptor accessory protein 5 | -1.262 | 6.07E-05 | 1419398_a_at | R |
| AATK | apoptosis-associated tyrosine kinase | -1.261 | 2.22E-05 | 1416936_at |  |
| HCK | hemopoietic cell kinase | -1.261 | 9.20E-06 | 1449455_at |  |
| LHX6 | LIM homeobox 6 | -1.260 | 1.92E-05 | 1425094_a_at | R |
| HIST1H2AE | histone cluster 1, H2ae | -1.259 | 4.65E-06 | 1438009_at |  |
| TNNC2 | troponin C type 2 (fast) | -1.259 | 6.24E-06 | 1417464_at |  |
| TYROBP | TYRO protein tyrosine kinase binding protein | -1.259 | 5.94E-06 | 1450792_at |  |
| ARHGEF3 | Rho guanine nucleotide exchange factor (GEF) 3 | -1.257 | 3.64E-06 | 1424250_a_at |  |
| HSD3B5 | hydroxy-delta-5-steroid dehydrogenase, 3 beta- and steroid delta-isomerase 5 | -1.257 | 2.08E-07 | 1420531_at |  |
| GMDS | GDP-mannose 4,6-dehydratase | -1.256 | 7.90E-06 | 1434158_at | R |
| LILRA6 | leukocyte immunoglobulin-like receptor, subfamily A (with TM domain), member 6 | -1.256 | 6.01E-06 | 1418809_at | R |
| CLEC6A | C-type lectin domain family 6, member A | -1.255 | 1.54E-06 | 1425951_a_at |  |
| RHOC | ras homolog gene family, member C | -1.255 | 7.51E-06 | 1448605_at |  |
| LRRC16A | leucine rich repeat containing 16A | -1.253 | 2.08E-05 | 1451804_a_at |  |
| ITGB2 | integrin, beta 2 (complement component 3 receptor 3 and 4 subunit) | -1.252 | 1.09E-05 | 1450678_at |  |
| CD14 | CD14 molecule | -1.251 | 4.70E-05 | 1417268_at |  |
| CD68 | CD68 molecule | -1.251 | 2.42E-05 | 1449164_at |  |
| CES5 | carboxylesterase 5 | -1.250 | 7.62E-05 | 1427137_at |  |
| LY86 | lymphocyte antigen 86 | -1.248 | 5.00E-05 | 1422903_at |  |
| CYP4A31 | cytochrome P450, family 4, subfamily a, polypeptide 31 | -1.247 | 1.42E-05 | 1424943_at |  |
| GPD2 | glycerol-3-phosphate dehydrogenase 2 (mitochondrial) | -1.247 | 1.29E-05 | 1452741_s_at | R |
| TSC22D1 | TSC22 domain family, member 1 | -1.246 | 5.34E-05 | 1433899_x_at | R |
| 4931408D14RIK | RIKEN cDNA 4931408D14 gene | -1.245 | 2.17E-04 | 1431806_at |  |
| NRP1 | neuropilin 1 | -1.245 | 5.89E-05 | 1448944_at | R |
| TXNIP | thioredoxin interacting protein | -1.245 | 3.31E-05 | 1415997_at |  |
| PTPRC | protein tyrosine phosphatase, receptor type, C | -1.244 | 4.76E-05 | 1422124_a_at |  |
| C1QC | complement component 1, q subcomponent, C chain | -1.243 | 8.04E-05 | 1449401_at |  |
| RAB30 | RAB30, member RAS oncogene family | -1.243 | 2.88E-05 | 1426452_a_at |  |
| SLC41A2 | solute carrier family 41, member 2 | -1.242 | 6.83E-05 | 1452445_at |  |
| ACOT1 | acyl-CoA thioesterase 1 | -1.241 | 1.15E-04 | 1422925_s_at |  |
| ASB2 | ankyrin repeat and SOCS box-containing 2 | -1.241 | 2.79E-05 | 1428444_at |  |
| CCND1 | cyclin D1 | -1.241 | 1.19E-04 | 1448698_at | R |
| GALE | UDP-galactose-4-epimerase | -1.240 | 1.63E-04 | 1424140_at |  |
| NUCB2 | nucleobindin 2 | -1.239 | 2.12E-05 | 1418355_at |  |
| Unknown | Unknown | -1.237 | 6.58E-05 | 1427820_at |  |
| EMR4P | egf-like module containing, mucin-like, hormone receptor-like 4 pseudogene | -1.235 | 1.48E-04 | 1451563_at | R |
| PTP4A3 | protein tyrosine phosphatase type IVA, member 3 | -1.235 | 6.54E-05 | 1418181_at |  |
| ARL5B | ADP-ribosylation factor-like 5B | -1.234 | 3.75E-05 | 1437884_at |  |
| TNNI2 | troponin I type 2 (skeletal, fast) | -1.234 | 5.02E-06 | 1416889_at |  |
| SEMA5B | sema domain, seven thrombospondin repeats (type 1 and type 1-like), transmembrane domain (TM) and short cytoplasmic domain, (semaphorin) 5B | -1.233 | 6.90E-05 | 1422146_at |  |
| LGALS1 | lectin, galactoside-binding, soluble, 1 | -1.232 | 1.99E-04 | 1455439_a_at | R |
| SDF2L1 | stromal cell-derived factor 2-like 1 | -1.232 | 1.80E-04 | 1418206_at |  |
| SLC15A3 | solute carrier family 15, member 3 | -1.231 | 1.02E-04 | 1420697_at |  |
| CYBA | cytochrome b-245, alpha polypeptide | -1.230 | 1.98E-04 | 1454268_a_at |  |
| FPR1 | formyl peptide receptor 1 | -1.230 | 2.27E-05 | 1450808_at |  |
| BCL6 | B-cell CLL/lymphoma 6 | -1.228 | 1.37E-04 | 1450381_a_at | R |
| CRELD2 | cysteine-rich with EGF-like domains 2 | -1.227 | 3.70E-04 | 1452754_at |  |
| NFKBIZ | nuclear factor of kappa light polypeptide gene enhancer in B-cells inhibitor, zeta | -1.227 | 1.32E-04 | 1417483_at |  |
| ACOX1 | acyl-Coenzyme A oxidase 1, palmitoyl | -1.226 | 6.17E-05 | 1444518_at |  |
| ORM3 | orosomucoid 3 | -1.226 | 7.46E-05 | 1450611_at |  |
| PFN2 | profilin 2 | -1.226 | 1.01E-04 | 1418209_a_at |  |
| CD44 | CD44 molecule (Indian blood group) | -1.225 | 1.66E-04 | 1423760_at |  |
| CXCL2 | chemokine (C-X-C motif) ligand 2 | -1.225 | 1.54E-04 | 1419209_at | R |
| THRSP | thyroid hormone responsive (SPOT14 homolog, rat) | -1.225 | 1.01E-05 | 1422973_a_at | R |
| SCARA5 | scavenger receptor class A, member 5 (putative) | -1.221 | 1.91E-04 | 1451204_at |  |
| ACTA1 | actin, alpha 1, skeletal muscle | -1.220 | 1.08E-04 | 1427735_a_at |  |
| SPRED1 | sprouty-related, EVH1 domain containing 1 | -1.220 | 1.37E-04 | 1428777_at |  |
| TOB1 | transducer of ERBB2, 1 | -1.220 | 1.61E-04 | 1440844_at |  |
| VSIG4 | V-set and immunoglobulin domain containing 4 | -1.219 | 6.13E-04 | 1451651_at |  |
| FPR2 | formyl peptide receptor 2 | -1.216 | 1.46E-04 | 1422953_at |  |
| NEDD4L | neural precursor cell expressed, developmentally down-regulated 4-like | -1.216 | 3.39E-04 | 1457374_at | R |
| SFRS2 | splicing factor, arginine/serine-rich 2 | -1.216 | 4.00E-04 | 1415807_s_at |  |
| CYP2D40 | cytochrome P450, family 2, subfamily d, polypeptide 40 | -1.215 | 2.99E-04 | 1430814_at |  |
| GPD1 | glycerol-3-phosphate dehydrogenase 1 (soluble) | -1.215 | 1.46E-04 | 1416204_at | R |
| PLA2G6 | phospholipase A2, group VI (cytosolic, calcium-independent) | -1.215 | 3.52E-04 | 1431278_s_at |  |
| CGREF1 | cell growth regulator with EF-hand domain 1 | -1.214 | 1.06E-04 | 1424528_at |  |
| SPRY4 | sprouty homolog 4 (Drosophila) | -1.214 | 7.99E-05 | 1445669_at |  |
| C1QB | complement component 1, q subcomponent, B chain | -1.213 | 3.06E-04 | 1434366_x_at | R |
| CES1 | carboxylesterase 1 | -1.213 | 3.77E-04 | 1449486_at |  |
| CLPX | ClpX caseinolytic peptidase X homolog (E. coli) | -1.213 | 2.83E-04 | 1439342_at |  |
| DYNLL1 | dynein, light chain, LC8-type 1 | -1.212 | 6.56E-04 | 1448682_at | R |
| NRP1 | neuropilin 1 | -1.212 | 5.89E-04 | 1448943_at | R |
| CD55 | CD55 molecule, decay accelerating factor for complement (Cromer blood group) | -1.211 | 8.10E-05 | 1460242_at |  |
| HSPA13 | heat shock protein 70kDa family, member 13 | -1.211 | 4.98E-04 | 1429502_at | R |
| NRP1 | neuropilin 1 | -1.211 | 8.54E-04 | 1457198_at | R |
| CD207 | CD207 molecule, langerin | -1.210 | 1.93E-04 | 1425243_at |  |
| CTLA2B | cytotoxic T lymphocyte-associated protein 2 beta | -1.210 | 2.60E-04 | 1452352_at |  |
| ITGAL | integrin, alpha L (antigen CD11A (p180), lymphocyte function-associated antigen 1; alpha polypeptide) | -1.210 | 4.45E-04 | 1435560_at |  |
| RETSAT | retinol saturase (all-trans-retinol 13,14-reductase) | -1.210 | 4.85E-04 | 1424716_at | R |
| ODF3B | outer dense fiber of sperm tails 3B | -1.209 | 3.44E-04 | 1443671_x_at |  |
| C1QB | complement component 1, q subcomponent, B chain | -1.208 | 6.95E-04 | 1437726_x_at | R |
| LGALS1 | lectin, galactoside-binding, soluble, 1 | -1.208 | 5.11E-04 | 1419573_a_at | R |
| LRG1 | leucine-rich alpha-2-glycoprotein 1 | -1.208 | 1.24E-10 | 1417290_at |  |
| Unknown | Unknown | -1.208 | 1.88E-04 | 1435144_at |  |
| LEPR | leptin receptor | -1.207 | 1.28E-04 | 1456156_at |  |
| NR1I2 | nuclear receptor subfamily 1, group I, member 2 | -1.207 | 7.01E-04 | 1425723_at |  |
| PFKFB3 | 6-phosphofructo-2-kinase/fructose-2,6-biphosphatase 3 | -1.207 | 4.97E-04 | 1416432_at |  |
| FGF1 | fibroblast growth factor 1 (acidic) | -1.204 | 7.04E-04 | 1423136_at |  |
| FNDC3B | fibronectin type III domain containing 3B | -1.204 | 5.54E-04 | 1433833_at |  |
| PTGR1 | prostaglandin reductase 1 | -1.204 | 3.96E-04 | 1417777_at |  |
| COL27A1 | collagen, type XXVII, alpha 1 | -1.203 | 8.35E-04 | 1453191_at | R |
| FERMT3 | fermitin family homolog 3 (Drosophila) | -1.203 | 8.19E-05 | 1433963_a_at |  |
| CRCT1 | cysteine-rich C-terminal 1 | -1.202 | 1.77E-04 | 1453092_at |  |
| EVL | Enah/Vasp-like | -1.202 | 6.74E-04 | 1434920_a_at |  |
| LHX6 | LIM homeobox 6 | -1.202 | 4.17E-04 | 1422262_a_at | R |
| CYBB | cytochrome b-245, beta polypeptide | -1.201 | 4.11E-04 | 1422978_at |  |
| DNAJC3 | DnaJ (Hsp40) homolog, subfamily C, member 3 | -1.201 | 9.08E-04 | 1419163_s_at |  |
| OSGIN1 | oxidative stress induced growth inhibitor 1 | -1.201 | 6.88E-04 | 1424022_at |  |
| GPR172B | G protein-coupled receptor 172B | -1.199 | 4.71E-04 | 1439451_x_at |  |
| CSF1R | colony stimulating factor 1 receptor | -1.198 | 4.83E-04 | 1419872_at |  |
| PRSS29 | protease, serine, 29 | -1.197 | 6.38E-04 | 1449992_at |  |
| RAC2 | ras-related C3 botulinum toxin substrate 2 (rho family, small GTP binding protein Rac2) | -1.197 | 9.69E-04 | 1417620_at |  |
| ABCG2 | ATP-binding cassette, sub-family G (WHITE), member 2 | -1.196 | 9.10E-04 | 1422906_at |  |
| SSR1 | signal sequence receptor, alpha | -1.196 | 5.81E-04 | 1448843_at | R |
| TMEM141 | transmembrane protein 141 | -1.196 | 4.33E-04 | 1435258_at | R |
| B3GALNT1 | beta-1,3-N-acetylgalactosaminyltransferase 1 (globoside blood group) | -1.194 | 2.22E-04 | 1418736_at |  |
| CTSC | cathepsin C | -1.194 | 2.30E-04 | 1437939_s_at |  |
| SETD4 | SET domain containing 4 | -1.193 | 8.19E-04 | 1460373_a_at |  |
| VAT1 | vesicle amine transport protein 1 homolog (T. californica) | -1.193 | 7.20E-04 | 1438165_x_at |  |
| D130062J21RIK | RIKEN cDNA D130062J21 gene | -1.192 | 8.04E-04 | 1446929_at |  |
| FMO5 | flavin containing monooxygenase 5 | -1.192 | 7.96E-04 | 1440899_at | R |
| SPIC | Spi-C transcription factor (Spi-1/PU.1 related) | -1.191 | 6.35E-04 | 1418555_x_at | R |
| Unknown | Unknown | -1.191 | 7.57E-04 | 1425738_at |  |
| 1700106J16RIK | RIKEN cDNA 1700106J16 gene | -1.190 | 4.36E-04 | 1431441_at |  |
| CYP4A22 | cytochrome P450, family 4, subfamily A, polypeptide 22 | -1.189 | 1.80E-04 | 1424352_at |  |
| THADA | thyroid adenoma associated | -1.189 | 5.57E-04 | 1457106_at |  |
| Unknown | Unknown | -1.189 | 6.55E-05 | 1446423_at |  |
| C3AR1 | complement component 3a receptor 1 | -1.188 | 6.95E-04 | 1442082_at |  |
| CDC25C | cell division cycle 25 homolog C (S. pombe) | -1.188 | 6.67E-04 | 1456077_x_at |  |
| PDLIM5 | PDZ and LIM domain 5 | -1.187 | 9.75E-04 | 1450786_x_at |  |
| FMO5 | flavin containing monooxygenase 5 | -1.185 | 3.91E-05 | 1450332_s_at | R |
| PID1 | phosphotyrosine interaction domain containing 1 | -1.185 | 5.16E-04 | 1444969_at |  |
| IRAK3 | interleukin-1 receptor-associated kinase 3 | -1.184 | 7.65E-04 | 1435040_at |  |
| LCP2 | lymphocyte cytosolic protein 2 (SH2 domain containing leukocyte protein of 76kDa) | -1.184 | 9.90E-04 | 1418641_at |  |
| PROX1 | prospero homeobox 1 | -1.184 | 7.75E-04 | 1437894_at |  |
| SLCO1A1 | solute carrier organic anion transporter family, member 1a1 | -1.184 | 1.10E-04 | 1420379_at |  |
| INSIG2 | insulin induced gene 2 | -1.180 | 6.85E-04 | 1417982_at | R |
| MRPS25 | mitochondrial ribosomal protein S25 | -1.178 | 8.68E-04 | 1418716_at |  |
| THRSP | thyroid hormone responsive (SPOT14 homolog, rat) | -1.176 | 4.51E-06 | 1424737_at | R |
| SERP1 | stress-associated endoplasmic reticulum protein 1 | -1.174 | 6.94E-04 | 1415828_a_at |  |
| TMSB4X | thymosin beta 4, X-linked | -1.171 | 4.64E-04 | 1415906_at |  |
| Unknown | Unknown | -1.162 | 9.23E-04 | 1442336_at |  |
| Unknown | Unknown | -1.160 | 5.10E-04 | 1444846_at |  |
| ACSL1 | acyl-CoA synthetase long-chain family member 1 | -1.146 | 6.04E-04 | 1422526_at |  |
| SPIC | Spi-C transcription factor (Spi-1/PU.1 related) | -1.146 | 3.22E-04 | 1449134_s_at | R |
| RETSAT | retinol saturase (all-trans-retinol 13,14-reductase) | -1.143 | 1.37E-04 | 1424715_at | R |
| GULP1 | GULP, engulfment adaptor PTB domain containing 1 | -1.134 | 4.59E-04 | 1434423_at |  |
| CYP2C70 | cytochrome P450, family 2, subfamily c, polypeptide 70 | -1.132 | 2.19E-04 | 1424273_at |  |
| SLC22A1 | solute carrier family 22 (organic cation transporter), member 1 | -1.132 | 7.26E-04 | 1418118_at |  |
| PLIN2 | perilipin 2 | -1.128 | 6.27E-06 | 1448318_at |  |
| CYP3A43 | cytochrome P450, family 3, subfamily A, polypeptide 43 | -1.108 | 1.00E-03 | 1424973_at |  |
| CA3 | carbonic anhydrase III, muscle specific | -1.100 | 1.95E-05 | 1460256_at | R |
| HBD | hemoglobin, delta | -1.100 | 4.29E-05 | 1417184_s_at |  |
| GNMT | glycine N-methyltransferase | 1.087 | 1.01E-04 | 1417422_at |  |
| SERPINA1 | serpin peptidase inhibitor, clade A (alpha-1 antiproteinase, antitrypsin), member 1 | 1.103 | 2.75E-05 | 1420553_x_at | R |
| CTSL2 | cathepsin L2 | 1.115 | 6.70E-04 | 1451310_a_at |  |
| FTL | ferritin, light polypeptide | 1.115 | 3.97E-04 | 1418364_a_at |  |
| MBL2 | mannose-binding lectin (protein C) 2, soluble (opsonic defect) | 1.129 | 2.75E-04 | 1418787_at |  |
| ZFAND6 | zinc finger, AN1-type domain 6 | 1.134 | 6.39E-04 | 1442236_at |  |
| CLCA4 | chloride channel accessory 4 | 1.135 | 4.22E-04 | 1447394_at |  |
| ARAF | v-raf murine sarcoma 3611 viral oncogene homolog | 1.144 | 8.06E-04 | 1428607_at |  |
| CYP4A14 | cytochrome P450, family 4, subfamily a, polypeptide 14 | 1.144 | 7.87E-04 | 1423257_at |  |
| SLC7A2 | solute carrier family 7 (cationic amino acid transporter, y+ system), member 2 | 1.150 | 4.38E-04 | 1436555_at | R |
| ESCO2 | establishment of cohesion 1 homolog 2 (S. cerevisiae) | 1.152 | 3.89E-04 | 1428304_at |  |
| PRKCZ2 | protein kinase C, zeta 2 | 1.152 | 5.73E-04 | 1443886_at |  |
| Unknown | Unknown | 1.152 | 4.49E-04 | 1459062_x_at |  |
| ASS1 | argininosuccinate synthetase 1 | 1.156 | 3.21E-11 | 1416239_at |  |
| TNNT2 | troponin T type 2 (cardiac) | 1.158 | 8.61E-06 | 1418726_a_at |  |
| ELOVL2 | elongation of very long chain fatty acids (FEN1/Elo2, SUR4/Elo3, yeast)-like 2 | 1.162 | 3.06E-04 | 1416444_at |  |
| Unknown | Unknown | 1.163 | 1.50E-04 | 1458757_at |  |
| CPSF3 | cleavage and polyadenylation specific factor 3, 73kDa | 1.166 | 7.80E-04 | 1437328_x_at |  |
| ARNTL2 | aryl hydrocarbon receptor nuclear translocator-like 2 | 1.173 | 9.50E-04 | 1429688_at |  |
| Unknown | Unknown | 1.179 | 7.31E-04 | 1459330_at |  |
| PTPRD | protein tyrosine phosphatase, receptor type, D | 1.182 | 7.37E-04 | 1445767_at |  |
| RBM39 | RNA binding motif protein 39 | 1.182 | 8.20E-04 | 1442744_at |  |
| AMIGO2 | adhesion molecule with Ig-like domain 2 | 1.185 | 8.86E-04 | 1434601_at |  |
| Unknown | Unknown | 1.185 | 4.35E-04 | 1437694_at |  |
| MBLAC2 | metallo-beta-lactamase domain containing 2 | 1.187 | 9.13E-04 | 1437261_at |  |
| PDCD2L | programmed cell death 2-like | 1.187 | 7.01E-04 | 1426844_a_at |  |
| Unknown | Unknown | 1.187 | 4.78E-04 | 1419779_at |  |
| SERPINA1 | serpin peptidase inhibitor, clade A (alpha-1 antiproteinase, antitrypsin), member 1 | 1.188 | 6.65E-04 | 1449321_x_at | R |
| SRR | serine racemase | 1.188 | 8.05E-04 | 1438550_x_at |  |
| FLCN | folliculin | 1.190 | 7.00E-04 | 1456744_x_at |  |
| DHCR24 | 24-dehydrocholesterol reductase | 1.191 | 2.43E-04 | 1451895_a_at |  |
| GABARAPL1 | GABA(A) receptor-associated protein like 1 | 1.193 | 9.39E-05 | 1416418_at | R |
| CTH | cystathionase (cystathionine gamma-lyase) | 1.195 | 8.58E-06 | 1426243_at |  |
| HIVEP2 | human immunodeficiency virus type I enhancer binding protein 2 | 1.195 | 8.26E-04 | 1422018_at |  |
| A630035D09RIK | RIKEN cDNA A630035D09 gene | 1.196 | 4.31E-04 | 1441464_at |  |
| MAGI1 | membrane associated guanylate kinase, WW and PDZ domain containing 1 | 1.196 | 9.94E-04 | 1451893_s_at |  |
| ARHGAP6 | Rho GTPase activating protein 6 | 1.198 | 3.47E-05 | 1417704_a_at | R |
| EG668525 | predicted gene, EG668525 | 1.198 | 6.75E-04 | 1449651_x_at |  |
| RNPC3 | RNA-binding region (RNP1, RRM) containing 3 | 1.199 | 5.44E-04 | 1437461_s_at |  |
| CAMK1D | calcium/calmodulin-dependent protein kinase ID | 1.203 | 3.03E-04 | 1438643_at | R |
| LPIN1 | lipin 1 | 1.203 | 1.38E-04 | 1426516_a_at | R |
| TOB2 | transducer of ERBB2, 2 | 1.205 | 5.23E-04 | 1448666_s_at |  |
| CCDC41 | coiled-coil domain containing 41 | 1.206 | 3.51E-04 | 1453045_at |  |
| ZNF235 | zinc finger protein 235 | 1.207 | 2.33E-04 | 1429988_at |  |
| SLC7A2 | solute carrier family 7 (cationic amino acid transporter, y+ system), member 2 | 1.209 | 7.89E-04 | 1426008_a_at | R |
| TMEM97 | transmembrane protein 97 | 1.209 | 8.69E-04 | 1416376_at |  |
| AKR1C19 | aldo-keto reductase family 1, member C19 | 1.210 | 4.38E-04 | 1455454_at |  |
| FUBP1 | far upstream element (FUSE) binding protein 1 | 1.210 | 2.73E-04 | 1433482_a_at |  |
| Unknown | Unknown | 1.210 | 3.42E-04 | 1457141_at |  |
| Unknown | Unknown | 1.211 | 3.35E-04 | 1435628_x_at |  |
| IVD | isovaleryl Coenzyme A dehydrogenase | 1.212 | 4.45E-04 | 1449001_at |  |
| UPP2 | uridine phosphorylase 2 | 1.212 | 2.59E-06 | 1424969_s_at | R |
| AHNAK | AHNAK nucleoprotein | 1.213 | 1.81E-04 | 1428057_a_at |  |
| CAMK1D | calcium/calmodulin-dependent protein kinase ID | 1.215 | 9.44E-04 | 1452050_at | R |
| RGS3 | regulator of G-protein signaling 3 | 1.215 | 7.91E-05 | 1425701_a_at |  |
| NUPL1 | nucleoporin like 1 | 1.217 | 3.42E-04 | 1437843_s_at |  |
| PLK3 | polo-like kinase 3 (Drosophila) | 1.217 | 4.68E-04 | 1434496_at |  |
| ACPL2 | acid phosphatase-like 2 | 1.219 | 1.34E-04 | 1456735_x_at |  |
| NEDD9 | neural precursor cell expressed, developmentally down-regulated 9 | 1.219 | 2.84E-04 | 1447885_x_at |  |
| LPIN1 | lipin 1 | 1.220 | 3.15E-05 | 1418288_at | R |
| Unknown | Unknown | 1.220 | 3.70E-04 | 1445562_at |  |
| ALAS1 | aminolevulinate, delta-, synthase 1 | 1.222 | 4.02E-04 | 1455282_x_at | R |
| CRTC2 | CREB regulated transcription coactivator 2 | 1.222 | 2.46E-04 | 1429373_x_at |  |
| C16ORF68 | chromosome 16 open reading frame 68 | 1.223 | 7.29E-05 | 1439425_x_at |  |
| DPYD | dihydropyrimidine dehydrogenase | 1.223 | 3.59E-05 | 1427945_at |  |
| TGTP | T-cell specific GTPase | 1.223 | 4.15E-04 | 1449009_at |  |
| Unknown | Unknown | 1.223 | 4.16E-04 | 1443547_at |  |
| XLR4B | X-linked lymphocyte-regulated 4B | 1.223 | 5.02E-05 | 1449347_a_at |  |
| OAT | ornithine aminotransferase (gyrate atrophy) | 1.224 | 1.75E-11 | 1416452_at |  |
| Unknown | Unknown | 1.224 | 7.21E-04 | 1447699_at |  |
| ZAP70 | zeta-chain (TCR) associated protein kinase 70kDa | 1.224 | 1.30E-04 | 1422701_at |  |
| 3110052M02RIK | RIKEN cDNA 3110052M02 gene | 1.225 | 4.96E-05 | 1453668_at |  |
| CORO1C | coronin, actin binding protein, 1C | 1.225 | 2.93E-04 | 1437721_at |  |
| DCLK3 | doublecortin-like kinase 3 | 1.226 | 3.82E-05 | 1436532_at |  |
| GAS2L3 | growth arrest-specific 2 like 3 | 1.226 | 5.52E-05 | 1455980_a_at |  |
| MTHFD1 | methylenetetrahydrofolate dehydrogenase (NADP+ dependent) 1, methenyltetrahydrofolate cyclohydrolase, formyltetrahydrofolate synthetase | 1.227 | 2.04E-04 | 1436704_x_at |  |
| PCBP4 | poly(rC) binding protein 4 | 1.227 | 1.08E-04 | 1433658_x_at |  |
| GSN | gelsolin (amyloidosis, Finnish type) | 1.228 | 7.86E-05 | 1456312_x_at | R |
| BCAS2 | breast carcinoma amplified sequence 2 | 1.229 | 1.03E-04 | 1437262_x_at |  |
| ADORA1 | adenosine A1 receptor | 1.230 | 8.81E-05 | 1435495_at |  |
| PRKD3 | protein kinase D3 | 1.230 | 3.81E-04 | 1444691_at |  |
| ZWINT | ZW10 interactor | 1.230 | 3.85E-05 | 1427539_a_at |  |
| IRGM2 | immunity-related GTPase family M member 2 | 1.232 | 1.43E-04 | 1417793_at |  |
| Unknown | Unknown | 1.234 | 9.60E-05 | 1443138_at |  |
| WDR18 | WD repeat domain 18 | 1.235 | 1.10E-04 | 1443758_at |  |
| 1700049G17RIK | RIKEN cDNA 1700049G17 gene | 1.236 | 3.25E-05 | 1440171_x_at |  |
| ACP1 | acid phosphatase 1, soluble | 1.236 | 5.26E-05 | 1422716_a_at |  |
| GVIN1 | GTPase, very large interferon inducible 1 | 1.237 | 5.91E-05 | 1429184_at |  |
| HMGCR | 3-hydroxy-3-methylglutaryl-Coenzyme A reductase | 1.238 | 4.12E-05 | 1427229_at |  |
| ATR | ataxia telangiectasia and Rad3 related | 1.239 | 2.69E-05 | 1438921_at |  |
| ZNF808 | zinc finger protein 808 | 1.239 | 1.10E-04 | 1449972_s_at |  |
| HIVEP1 | human immunodeficiency virus type I enhancer binding protein 1 | 1.241 | 3.86E-05 | 1422742_at |  |
| VLDLR | very low density lipoprotein receptor | 1.242 | 1.55E-06 | 1417900_a_at |  |
| RAB14 | RAB14, member RAS oncogene family | 1.243 | 4.04E-05 | 1419245_at |  |
| C4ORF43 | chromosome 4 open reading frame 43 | 1.245 | 3.53E-05 | 1431117_x_at |  |
| MBD1 | methyl-CpG binding domain protein 1 | 1.246 | 5.88E-06 | 1453678_at |  |
| SLC7A2 | solute carrier family 7 (cationic amino acid transporter, y+ system), member 2 | 1.247 | 9.05E-05 | 1422648_at | R |
| TSPAN7 | tetraspanin 7 | 1.249 | 2.02E-05 | 1459812_x_at |  |
| ZSCAN21 | zinc finger and SCAN domain containing 21 | 1.249 | 1.32E-05 | 1449732_at |  |
| AGXT | alanine-glyoxylate aminotransferase | 1.251 | 4.26E-07 | 1418833_at |  |
| HHEX | hematopoietically expressed homeobox | 1.251 | 7.32E-05 | 1423319_at |  |
| FADS1 | fatty acid desaturase 1 | 1.253 | 9.70E-08 | 1423680_at |  |
| Unknown | Unknown | 1.253 | 1.73E-07 | 1442879_at |  |
| ARHGAP6 | Rho GTPase activating protein 6 | 1.256 | 1.14E-05 | 1451867_x_at | R |
| KIAA0467 | KIAA0467 | 1.259 | 1.06E-05 | 1440819_s_at |  |
| GCK | glucokinase (hexokinase 4) | 1.260 | 4.40E-05 | 1425303_at | R |
| GAS2 | growth arrest-specific 2 | 1.261 | 1.71E-05 | 1450112_a_at |  |
| TMEM191C | transmembrane protein 191C | 1.262 | 1.09E-05 | 1420191_s_at |  |
| ALAS1 | aminolevulinate, delta-, synthase 1 | 1.263 | 7.16E-09 | 1424126_at | R |
| CYP39A1 | cytochrome P450, family 39, subfamily A, polypeptide 1 | 1.264 | 4.49E-05 | 1418780_at |  |
| PIGO | phosphatidylinositol glycan anchor biosynthesis, class O | 1.264 | 2.51E-05 | 1437142_a_at |  |
| Unknown | Unknown | 1.265 | 3.40E-05 | 1452426_x_at |  |
| CREB1 | cAMP responsive element binding protein 1 | 1.268 | 5.86E-06 | 1421583_at |  |
| CLEC2D | C-type lectin domain family 2, member D | 1.269 | 7.85E-06 | 1424673_at | R |
| APOL9B | apolipoprotein L 9b | 1.273 | 9.43E-06 | 1424518_at |  |
| GSN | gelsolin (amyloidosis, Finnish type) | 1.275 | 2.58E-06 | 1437171_x_at | R |
| PMVK | phosphomevalonate kinase | 1.275 | 8.69E-06 | 1427893_a_at |  |
| TERT | telomerase reverse transcriptase | 1.276 | 9.00E-08 | 1456941_at |  |
| Unknown | Unknown | 1.276 | 4.67E-06 | 1458314_at |  |
| GOT1 | glutamic-oxaloacetic transaminase 1, soluble (aspartate aminotransferase 1) | 1.278 | 2.48E-08 | 1450970_at |  |
| FDPS | farnesyl diphosphate synthase (farnesyl pyrophosphate synthetase, dimethylallyltranstransferase, geranyltranstransferase) | 1.281 | 1.00E-06 | 1423418_at |  |
| SLC22A9 | solute carrier family 22 (organic anion transporter), member 9 | 1.283 | 9.13E-07 | 1451635_at |  |
| Unknown | Unknown | 1.286 | 1.20E-06 | 1426936_at |  |
| RDH11 | retinol dehydrogenase 11 (all-trans/9-cis/11-cis) | 1.290 | 6.84E-06 | 1449209_a_at | R |
| GSN | gelsolin (amyloidosis, Finnish type) | 1.296 | 3.68E-07 | 1436991_x_at | R |
| UPP2 | uridine phosphorylase 2 | 1.296 | 3.56E-07 | 1451548_at | R |
| Unknown | Unknown | 1.297 | 4.64E-07 | 1420292_x_at |  |
| NSDHL | NAD(P) dependent steroid dehydrogenase-like | 1.299 | 3.67E-06 | 1416222_at |  |
| TM7SF2 | transmembrane 7 superfamily member 2 | 1.299 | 1.88E-06 | 1460684_at |  |
| TP53BP1 | tumor protein p53 binding protein 1 | 1.301 | 5.38E-07 | 1426956_a_at |  |
| DHCR7 | 7-dehydrocholesterol reductase | 1.305 | 1.75E-06 | 1448619_at |  |
| PIR | pirin (iron-binding nuclear protein) | 1.307 | 2.54E-07 | 1429001_at |  |
| GCK | glucokinase (hexokinase 4) | 1.314 | 3.95E-07 | 1419146_a_at | R |
| MBP | myelin basic protein | 1.316 | 6.61E-08 | 1433532_a_at |  |
| IGHA1 | immunoglobulin heavy constant alpha 1 | 1.320 | 1.12E-07 | 1452574_x_at |  |
| HAMP | hepcidin antimicrobial peptide | 1.321 | 1.59E-10 | 1436643_x_at | R |
| MMD2 | monocyte to macrophage differentiation-associated 2 | 1.330 | 3.75E-08 | 1438654_x_at |  |
| Unknown | Unknown | 1.332 | 2.03E-08 | 1445062_at |  |
| SQLE | squalene epoxidase | 1.334 | 9.55E-07 | 1415993_at |  |
| AGXT2L1 | alanine-glyoxylate aminotransferase 2-like 1 | 1.335 | 2.64E-08 | 1431406_at | R |
| ALDH1B1 | aldehyde dehydrogenase 1 family, member B1 | 1.342 | 4.61E-08 | 1451260_at |  |
| MSI2 | musashi homolog 2 (Drosophila) | 1.342 | 7.88E-08 | 1435521_at |  |
| GLS2 | glutaminase 2 (liver, mitochondrial) | 1.350 | 2.94E-11 | 1435245_at |  |
| Unknown | Unknown | 1.351 | 2.85E-08 | 1441430_at |  |
| CYP51A1 | cytochrome P450, family 51, subfamily A, polypeptide 1 | 1.355 | 4.89E-09 | 1422533_at | R |
| Unknown | Unknown | 1.365 | 8.92E-11 | 1420291_at |  |
| RDH11 | retinol dehydrogenase 11 (all-trans/9-cis/11-cis) | 1.367 | 7.20E-09 | 1418760_at | R |
| KCND2 | potassium voltage-gated channel, Shal-related subfamily, member 2 | 1.372 | 4.38E-09 | 1447764_at |  |
| Unknown | Unknown | 1.375 | 4.48E-09 | 1455869_at |  |
| 9130409I23RIK | RIKEN cDNA 9130409I23 gene | 1.384 | 6.15E-09 | 1457403_at |  |
| NNMT | nicotinamide N-methyltransferase | 1.417 | 1.27E-11 | 1432517_a_at |  |
| CYP8B1 | cytochrome P450, family 8, subfamily B, polypeptide 1 | 1.428 | 4.47E-20 | 1449309_at |  |
| ONECUT1 | one cut homeobox 1 | 1.443 | 1.49E-10 | 1450252_at | R |
| AGXT2L1 | alanine-glyoxylate aminotransferase 2-like 1 | 1.448 | 4.73E-13 | 1452975_at | R |
| ONECUT1 | one cut homeobox 1 | 1.454 | 4.15E-12 | 1421447_at | R |
| Unknown | Unknown | 1.456 | 2.64E-12 | 1447698_x_at |  |
| SC4MOL | sterol-C4-methyl oxidase-like | 1.480 | 4.02E-12 | 1423078_a_at |  |
| CYP51A1 | cytochrome P450, family 51, subfamily A, polypeptide 1 | 1.515 | 2.82E-13 | 1450646_at | R |
| CYP1A1 | cytochrome P450, family 1, subfamily A, polypeptide 1 | 1.548 | 7.37E-17 | 1422217_a_at |  |
| CYP17A1 | cytochrome P450, family 17, subfamily A, polypeptide 1 | 1.582 | 1.56E-17 | 1417017_at |  |
| GSTA5 | glutathione S-transferase alpha 5 | 1.599 | 5.82E-17 | 1421040_a_at | R |
| BTN1A1 | butyrophilin, subfamily 1, member A1 | 1.623 | 9.61E-17 | 1425754_a_at |  |
| GSTA5 | glutathione S-transferase alpha 5 | 1.647 | 1.25E-17 | 1421041_s_at | R |
| IDI1 | isopentenyl-diphosphate delta isomerase 1 | 1.693 | 7.53E-20 | 1451122_at | R |
| IDI1 | isopentenyl-diphosphate delta isomerase 1 | 1.930 | 3.77E-34 | 1423804_a_at | R |
| CYP7A1 | cytochrome P450, family 7, subfamily A, polypeptide 1 | 1.971 | 5.92E-40 | 1438743_at | R |
| CYP7A1 | cytochrome P450, family 7, subfamily A, polypeptide 1 | 2.075 | 1.51E-40 | 1422100_at | R |

All differentially expressed transcripts (p<0.001) resulting from a comparison of H-P/C-HF with L-P/C-HF mice after 4-wk of feeding are listed. The significance of differences was estimated by a moderated ANOVA as described in the Material and Method section. The fold change with a negative and a positive value indicates down-regulation and up-regulation in the H-P/C-HF group, respectively. An Affymetrix probeset ID (Mouse 430 2.0) is provided for each gene. R in Notes represents a replicated detection with a different probeset for a given gene on the microarray
