# Supplementary material for: Consequences of Exchanging Carbohydrates for Proteins in the Cholesterol Metabolism of Mice Fed a High-fat Diet
Source: PLoS One. 2012 Nov 6;7(11):e49058. doi: 10.1371/journal.pone.0049058 (PMC3490911; doi:10.1371/journal.pone.0049058)
Supplement: Table S5 — Transcription factor analysis of global gene expressions at 4-wk after H-P/C-HF feeding. (DOC) [file pone.0049058.s008.doc]

**Table S5. Transcription factor analysis of global gene expressions at 4-wk after H-P/C-HF feeding**

| **Upstream Regulator** | **Fold Change** | **Molecule Type** | **Predicted Activation State** | **p-value of overlap** | **Target molecules in dataset** |
| --- | --- | --- | --- | --- | --- |
| SREBF2 |  | transcription regulator | Activated | 8.19E-15 | ACSL1, CAMK1D, CYP51A1, CYP8B1, DHCR7, FDPS, HMGCR, IDI1, MSMO1, NSDHL |
| SREBF1 (includes EG:176574) |  | transcription regulator | Activated | 2.84E-14 | ACSL1, CAMK1D, CFD, CYP51A1, CYP7A1, CYP8B1, DHCR7, FADS1, FDPS, GCK |
| SIRT2 |  | transcription regulator | Activated | 2.17E-06 | DHCR7, HMGCR, IDI1, PMVK, SQLE |
| FOXO1 |  | transcription regulator | Activated | 5.02E-05 | AGXT, ALAS1, CCND1, CTSL2, CYP7A1, FDPS, GCK, GPD1, GPD2, HMGCR |
| EGR2 |  | transcription regulator | Activated | 1.62E-03 | ASS1, CSF1R, CYP51A1, DHCR7, EGR1, HMGCR, ID2, MBP |
| PPARGC1B |  | transcription regulator | Inhibited | 4.05E-03 | DHCR24, FDPS, HMGCR, SQLE |
| PPARA |  | ligand-dependent nuclear receptor | Inhibited | 5.10E-21 | ACOX1, ACSL1, ACTA1, AGXT, ASS1, C1QA, C1QB, CCND1, CTH, CYBA |
| CEBPE |  | transcription regulator | Inhibited | 3.25E-05 | CD14, CSF1R, CTSL2, LCN2, Lyz1/Lyz2, MBP, ORM1/ORM2 |
| HMGA1 |  | transcription regulator | Inhibited | 8.33E-05 | CCND1, CD44 (includes EG:100330801), CTSC, CYP7A1, DHCR7, EGR1, GSN, HCK, HMGCR, IDI1 |
| WT1 |  | transcription regulator | Inhibited | 7.61E-04 | CCND1, CSF1R, CTSL2, EGR1, FDPS, FGF1, IDI1, SQLE, TERT |
| CLOCK |  | transcription regulator | Inhibited | 6.21E-02 | ACOX1, CYP51A1, GCK, LPIN1, NR0B2, SRR |
