# Supplementary material for: Consequences of Exchanging Carbohydrates for Proteins in the Cholesterol Metabolism of Mice Fed a High-fat Diet
Source: PLoS One. 2012 Nov 6;7(11):e49058. doi: 10.1371/journal.pone.0049058 (PMC3490911; doi:10.1371/journal.pone.0049058)
Supplement: Table S6 — Nutritional composition of the diets. (DOC) [file pone.0049058.s009.doc]

Table S6. Nutritional composition of the diets

|  | *L-P/C-HF* | | *H-P/C-HF* | |
| --- | --- | --- | --- | --- |
|  | Energy (%) | Wt (%) | Energy (%) | Wt (%) |
| Protein | 15 | 20.3 | 30 | 40.55 |
| Carbohydrate | 30 | 40.55 | 15 | 20.3 |
| Fat | 55 | 30.74 | 55 | 30.74 |
| Calorie (kcal/g) | 5.16 | | 5.24 | |

| *Wt (%)* | *L-P/C-HF* | *H-P/C-HF* |
| --- | --- | --- |
| Casein | 20.4 | 40.8 |
| L-Cystine | 0.2 | 0.4 |
| Maltodextrin 10 | 15.9 | 15.9 |
| Corn starch | 25.3 | 4.7 |
| Corn oil | 2.3 | 2.3 |
| Lard | 31.2 | 31.2 |
| Mineral mix AIN-93M | 3.5 | 3.5 |
| Vitamin mix AIN-93M | 1 | 1 |
| Choline bitartrate | 0.2 | 0.2 |
