# Supplementary material for: Consequences of Exchanging Carbohydrates for Proteins in the Cholesterol Metabolism of Mice Fed a High-fat Diet
Source: PLoS One. 2012 Nov 6;7(11):e49058. doi: 10.1371/journal.pone.0049058 (PMC3490911; doi:10.1371/journal.pone.0049058)
Supplement: Table S7 — Arrays were used to perform the LOO analysis for cross validation of the PCA. (DOC) [file pone.0049058.s010.doc]

Table S7. Arrays were used to perform the LOO analysis for cross validation of the PCA.

| **Leave out** | Comp 1 | Comp 2 | Comp 3 | Comp 4 | Comp 5 |
| --- | --- | --- | --- | --- | --- |
| All | 0.4304 | 0.3016 | 0.1553 | 0.0425 | 0.0264 |
| A01 | 0.4235 | 0.3163 | 0.1512 | 0.0375 | 0.0280 |
| A09 | 0.4135 | 0.2916 | 0.1752 | 0.0451 | 0.0317 |
| A24 | 0.4244 | 0.3076 | 0.1497 | 0.0473 | 0.0232 |
| B25 | 0.3912 | 0.3137 | 0.1774 | 0.0462 | 0.0228 |
| A22 | 0.4312 | 0.3049 | 0.1544 | 0.0446 | 0.0216 |
