# Supplementary material for: Consequences of Exchanging Carbohydrates for Proteins in the Cholesterol Metabolism of Mice Fed a High-fat Diet
Source: PLoS One. 2012 Nov 6;7(11):e49058. doi: 10.1371/journal.pone.0049058 (PMC3490911; doi:10.1371/journal.pone.0049058)
Supplement: Table S8 — A one-way ANOVA was applied to identify the outlier which significantly reduced the overall variability. B25 reduced the variability down to 61.9% of the initial variance. (DOC) [file pone.0049058.s011.doc]

Table S8. A one-way ANOVA was applied to identify the outlier which significantly reduced the overall variability. B25 reduced the variability down to 61.9 % of the initial variance.

| *Groups* | *Count* | *Sum* | *Average* | *Variance* | *%* |
| --- | --- | --- | --- | --- | --- |
| All | 3 | 0.8872 | 0.2957 | 0.0189 | 100.0 |
| A01 | 3 | 0.8910 | 0.2970 | 0.0188 | 99.3 |
| A09 | 3 | 0.8803 | 0.2934 | 0.0142 | 74.9 |
| A24 | 3 | 0.8817 | 0.2939 | 0.0190 | 100.3 |
| B25 | 3 | 0.8823 | 0.2941 | 0.0117 | 61.9 |
| A22 | 3 | 0.8905 | 0.2968 | 0.0192 | 101.4 |
