# Supplementary material for: Consequences of Exchanging Carbohydrates for Proteins in the Cholesterol Metabolism of Mice Fed a High-fat Diet
Source: PLoS One. 2012 Nov 6;7(11):e49058. doi: 10.1371/journal.pone.0049058 (PMC3490911; doi:10.1371/journal.pone.0049058)
Supplement: Text S1 — (DOC) [file pone.0049058.s012.doc]

**Supporting information**

**Quality control of Affymetrix chips**

The quality of raw data from Affymetrix chips was examined before further analysis. Because the data were normally distributed, a Pearson’s Product Moment Correlation was applied to identify atypical observations. A01, A09, A24, and B25 showed lower correlation coefficients, and were therefore identified to be potential outliers (Figure S1). The data matrix was further clustered in order to identify groups of arrays with an atypical behavior (Figure S2).

**Principal component analysis (PCA) and Leave-one-out (LOO) cross validation**

The PCA was performed on the basis of the entire probeset. Our results show that approximate 88% of the entire variance could be explained by the first 3 components (Figure S2). The PCA analysis was validated using a leave-one-out (LOO) method. The 4 putative outliers described above were separately removed from the second PCA analysis, and a similar contribution of the first 3 components to the overall variance would be expected (Figure S3, Table S7).

The variability among the first 3 components was tested by one-way ANOVA. A randomly chosen array, A22, was left out as a positive control, the absence of which only marginally improves the overall variance. In contrast, removing B25 from the data matrix decreased the initial variability by nearly 40% (Table S8). We therefore declared that B25 was an outlier, and this array was removed from the final analysis.

**Supplementary Information Legends**

Figure S1. Quality control of Affymetrix chips. Intensity plot of Pearson’s product correlation matrix.

Figure S2. Cluster analysis of the intensity plot, whereas B25, A24, A09, and A01 were grouped in two sub-clusters, separated from the other datasets, respectively.

Figure S3. Principal component analysis (PCA) and Leave-one-out (LOO) cross validation

The contributions to the overall variance are plotted for each putative outlier and for the first 5 components respectively.

Table S1. Organ weight of mice at sacrifice. Data are median ± SE, n=12/group. ND, not determined.

Table S2. List of regulated transcripts after H-P/C-HF feeding for 2 days. All differentially expressed transcripts (p<0.001) resulting from a comparison of H-P/C-HF with L-P/C-HF mice after 2-d of feeding are listed. The significance of differences was estimated by a moderated ANOVA as described in the Material and Method section. The fold change with a negative and a positive value indicates down-regulation and up-regulation in the H-P/C-HF group, respectively. An Affymetrix probeset ID (Mouse 430 2.0) is provided for each gene. R in Notes represents a replicated detection with an alternative probeset for a given gene on the microarray.

Table S3. Transcription factor analysis of global gene expressions at 2-d after H-P/C-HF feeding.

Table S4. List of regulated transcripts after H-P/C-HF feeding for 4 wks. All differentially expressed transcripts (p<0.001) resulting from a comparison of H-P/C-HF with L-P/C-HF mice after 4-wk of feeding are listed. The significance of differences was estimated by a moderated ANOVA as described in the Material and Method section. The fold change with a negative and a positive value indicates down-regulation and up-regulation in the H-P/C-HF group, respectively. An Affymetrix probeset ID (Mouse 430 2.0) is provided for each gene. R in Notes represents a replicated detection with a different probeset for a given gene on the microarray.

Table S5. Transcription factor analysis of global gene expressions at 4-wk after H-P/C-HF feeding

Table S6. Nutritional composition of the diets

Table S7. Arrays were used to perform the LOO analysis for cross validation of the PCA.

Table S8. A one-way ANOVA was applied to identify the outlier which significantly reduced the overall variability. B25 reduced the variability down to 61.9 % of the initial variance.
